# The thyroid hormone activating enzyme, DIO2, is a potential pan-cancer biomarker and immunotherapy target

**DOI:** 10.1007/s40618-024-02526-9

**Published:** 2025-01-17

**Authors:** A. Nappi, C. Miro, A. G. Cicatiello, S. Sagliocchi, L. Acampora, F. Restolfer, M. Dentice

**Affiliations:** 1 Department of Clinical Medicine and Surgery, University of Naples “Federico II”, 80131 Naples, Italy; 2 CEINGE–, Biotecnologie Avanzate S.c.a.r.l., 80131 Naples, Italy

**Keywords:** DIO2, Pan-cancer, Immune microenvironment, Genetic alternations, Clinical features

## Abstract

**Purpose:**

Type 2 deiodinase (D2), encoded by DIO2 gene, catalyzes the activation of the prohormone thyroxine (T4) into the bioactive hormone triiodothyronine (T3) in peripheral tissues, thereby regulating the intracellular Thyroid Hormone (TH) availability. Recently, several studies have demonstrated that a drastic increase in the peripheral activation of TH, via D2, fosters tumor progression, metastasis, and immunity.

**Methods:**

To further prove the clinical relevance of D2 in human cancer, based on public Database of The Cancer Genome Atlas (TCGA), we conducted a pan-cancer analysis of DIO2 expression in various cancer types and investigated the association of DIO2 expression with the tumor microenvironment (TME) components and immune cell infiltration, along with the DIO2 genetic alteration types.

**Results:**

Although with different expression levels between the various cancer types, the pan-cancer analysis showed that DIO2 was highly expressed in most tumors and related to the progression of some tumor types. Furthermore, DIO2 expression was also significantly correlated with TME components, immune cell infiltration, and immunoinhibitory and immunostimulatory gene subsets.

**Conclusion:**

The relevance of this study is that it adds a clinical relevance to the recent demonstrations that D2 accelerates tumor invasion in animal models and poses DIO2 gene as a potential prognostic marker in various human cancers.

**Supplementary Information:**

The online version contains supplementary material available at 10.1007/s40618-024-02526-9.

## Introduction

Thyroid Hormone (TH) plays an essential role in regulating countless pathophysiological processes in humans [1–3]. Secreted from the thyroid gland mainly in form of hormone precursor (thyroxine, T4), TH is activated into the bioactive hormone (triiodothyronine, T3) through a tight regulation of two TH‐activating enzymes, type I (D1, encoded by DIO1 gene) and type II (D2, encoded by DIO2 gene) deiodinases, that catalyze the conversion of T4 into T3. TH clearance is mainly based on the enzymatic activity of type III (D3, encoded by DIO3 gene) deiodinase, that removes an iodine moiety from both, T3 and T4, producing inactive metabolites [4]. Based on its tissue distribution and enzymatic properties, DIO2 is currently believed to be the main DIO responsible for activating TH in the body [5]. Moreover, research in animal models has demonstrated that altered peripheral control of the TH action greatly affects cancer proliferation, invasiveness, and angiogenesis [6–8]. The complex control of cancer formation and progression involves both the genomic and non-genomic action of TH [9]. Similarly, alterations in all the three deiodinases have been associated to cancer formation and progression [10–13]. Although conflicting results have been demonstrated on the clinical association between TH and cancer, a major piece of evidence indicates that subclinical and clinical hyperthyroidism increases the risk of several solid malignancies and aggravates the onset of cancer [14]. The role of the deiodinases in cancer is complex and still not completely understood. For instance, in a model of “benign” skin cancer as the Basal Cell Carcinoma (BCC), the most abundantly expressed deiodinase is D3 and its inhibition reduces BCC growth [15–17]. Also, D2 is expressed in the BCC and its down-regulation promotes cell proliferation [11]. Thus, in the BCC, D2-mediated TH activation is responsible for attenuation of cancer growth. However, the BCC has a low invasive and metastatic propension [18], and is not a proper model to address the progression and evolution of cancer cells toward malignancy. A different picture emerged from the study of the Squamous Cell Carcinoma (SCC), a keratinocyte-derided tumor that, compared to the BCC, has a relative higher metastatic potential [19]. Indeed, our group demonstrated that in a model of skin cancer progression, namely the chemical carcinogenesis-induced SCC, enabling to follow the multistep processing of tumor progression toward malignancy, D2 is expressed at late stages of SCC progression and its expression enhances the pro-metastatic evolution of SCC tumors [20].

In the SCC model we demonstrated that the D2-mediated TH activation increases the propensity to undergo Epithelial-to-Mesenchymal Transition (EMT), thereby enhancing the metastatic evolution of cancer. Besides the SCC, DIO2 has been shown to acts tumor promoter in different cancer types, such as bladder cancer [21], prostate cancer [22, 23], ovarian cancer [24], colon cancer [25] and skin cancer [8, 20], thus suggesting that DIO2 can be a valid oncogenic marker in different tumor types. Adding further support to this idea, we recently demonstrated that DIO2 expression and the consequent TH activation are induced by the loss of the oncosuppressor p53, which reinforce the concept that TH promotes evolution of cancer toward malignancy and that elevation of D2 levels is a key mark of advanced tumors [26].

Although the role of D2 in cancer is starting to be widely elucidated, nevertheless, the role of DIO2 as a prognostic marker in cancer or its epidemiological, clinical, and biostatistics relevance is still underestimated.

The innovation of the whole-genome sequencing has presented the opportunity to deeper analyze meta-analysis of genomic features across tumor types. These approaches offer the meaningful possibility to overcome the daunting heterogeneity of oncogenic cells and also meet the requirement of the precision medicine that is to offer patients targeted therapies based on genomics. From this point of view, the identification of novel biomarkers in cancer not only can be helpful for detection and diagnosis but might be essential to identify the best treatment options.

This study used pan-cancer data from The Cancer Genome Atlas (TCGA) to investigate the expression pattern of DIO2 and its association with primary Overall Survival (OS) in all the cancer types, and to correlate its expression level with the TME components, immune cell infiltration, immunomodulatory genes, and molecular pathways in various types of cancer, along with the types of DIO2 genetic alterations. The results of this study confirmed DIO2 as a potential biomarker in cancer and provide helpful information about the role of DIO2 in tumorigenesis since it extended our identification of DIO2 as a potential biomarker in multiple cancer types.

## Materials and methods

### Gene expression analysis

Input of DIO2 is made in the “Gene_DE” module of TIMER2.0 (Tumor IMmune Estimation Resource, version 2.0, http://timer.cistrome.org/) [27–29] and the DIO2 gene expression difference between tumor and adjacent normal tissues was analyzed for different tumors or specific tumor subtypes of the TCGA (The Cancer Genome Atlas) project. For tumor types without or with highly limited normal tissues, the “Expression Analysis-Box Plots” module of the GEPIA2 (Gene Expression Profiling Interactive Analysis, version 2, http://gepia2.cancer-pku.cn/#analysis) [30] was used to obtain the DIO2 expression difference between the these tumor tissues and the corresponding normal tissues of the GTEx (Genotype-Tissue Expression) Database, under the settings of p-value cutoff = 0.01, Log2FC (Fold Change) cutoff = 1, and “Match TCGA normal and GTEx data”. Furthermore, DIO2 gene expression in different pathological stages of all TCGA tumors was plotted as violin plots by the “Pathological Stage Plot” module of GEPIA2. The Log2 [TPM (Transcripts Per Million) + 1] transformed expression data were applied for the box or violin plots.

### Survival prognosis analysis

The “Survival Map” module of GEPIA2 (Gene Expression Profiling Interactive Analysis, version 2, http://gepia2.cancer-pku.cn/#analysis) [30] was used to obtain the Overall Survival (OS) and the Disease-Free Survival (DFS) data of DIO2 across all TCGA tumors. Cutoff-low (50%) and cutoff-high (50%) values were used as the expression thresholds for splitting the low- and high-expression cohorts. The Log-rank test was used in the hypothesis test.

### Genetic alteration analysis

After logging into the cBioPortal web (https://www.cbioportal.org/) [31, 32], in the Quickselect section was chosen the “TCGA Pan-Cancer Atlas Studies” and DIO2 gene was entered for queries of the genetic alteration characteristics. The results of the alteration frequency, mutation type and Copy Number Alteration (CNA) across all TCGA tumors were observed in the “Cancer Types Summary” module. The “Comparison” module was used to obtain the data on the Overall Survival, Disease-specific Survival, Disease-free Survival and Progression-free survival differences for the TCGA cancer cases with or without DIO2 genetic alteration. Kaplan–Meier (KM) plots with log-rank P-value were generated as well.

### Methylation promoter analysis

The UALCAN Database (The University of ALabama at Birmingham CANcer data analysis Portal, https://ualcan.path.uab.edu) [33] was used to evaluate the DIO2 promoter DNA methylation difference between tumor and adjacent normal tissues across all TCGA tumors and based on cancer stages (Illumina ID probes: cg16254394, TSS1500; cg07294774, TSS200, TSS1500; cg01555151, TSS1500; cg09191574, TSS1500; cg13033972, TSS1500; cg08183125, TSS1500).

### Immune infiltration analysis

The “Immune-Gene” module of the TIMER2.0 (Tumor IMmune Estimation Resource, version 2.0, http://timer.cistrome.org/) [27–29] was used to explore the association between DIO2 gene expression and immune infiltrates across all TCGA tumors. The Cancer-Associated Fibroblasts (CAFs), Dendritic Cells (DCs), Endothelial Cells (ECs), Tumor-Associated Macrophages (TAMs) and of CD4^+^ T-cells and CD8^+^ T-cells were selected. The EPIC, MCPCOUNTER, TIDE, XCELL and TIMER algorithms were applied for immune infiltration estimations. The P-values and partial correlation values were obtained via the purity-adjusted Spearman’s rank correlation test. The data were visualized as a heatmap.

### Immunogenomic analyses

TISIDB Database (http://cis.hku.hk/TISIDB/) [34] was used to analyze the correlation between DIO2 expression and immunoinhibitors, immunostimulators, Major Histocompatibility Complex (MHC) molecules, chemokines, and chemokine receptors in pan-cancer.

### DIO2‑related gene enrichment analysis

The “Expression Analysis-Similar Gene Detection” module of GEPIA2 (Gene Expression Profiling Interactive Analysis, version 2, http://gepia2.cancer-pku.cn/#analysis) [30] was used to obtain the top 100 DIO2-related target genes based on the datasets of all TCGA tumors and normal tissues. The “Expression Analysis-Correlation Analysis” module of GEPIA2 was used to perform a coupled gene Pearson correlation analysis of DIO2 and selected genes. The Log2 [TPM (Transcripts Per Million)] was applied for the dot-plot. Moreover, the “Exploration-Gene_Corr” module of TIMER2.0 (Tumor IMmune Estimation Resource, version 2.0, http://timer.cistrome.org/) [27–29] was used to obtain the heatmap data of the selected genes. Furthermore, based on twelve of the top 100 DIO2-correlated genes in TCGA project, Gene Ontology (GO) enrichment analysis in Biological Processes (BP) was performed by using the Enrichr suite of gene set enrichment analysis tool (https://maayanlab.cloud/Enrichr/) [35–37] and Metascape (https://metascape.org) [38]. GO enriched terms are visualized as GO chord and Volcano plot from the MSigDB_Hallmark_2020 gene set.

### Statistical analyses

DIO2 expression was compared between tumor and adjacent normal tissues employing the TCGA Database, with the results presented as p values, fold changes, and gene ranks. The survival results were presented as hazard ratios, 95% CI, and p values of Log-rank tests. For all statistical analyses, p < 0.05 was considered statistically significant.

## Results

### DIO2 Gene expression analysis in pan-cancer

In this study, we aimed to explore the oncogenic potential of the human DIO2 gene (Gene ID: 1734, https://www.ncbi.nlm.nih.gov/gene/1734, NM_000793.6 for mRNA) in pan-cancer. We applied the TIMER2.0 platform which represents a comprehensive tool for systematic analysis of differential gene expression profiles from TCGA Database, through the Wilcoxon test.

**Fig. 1:**
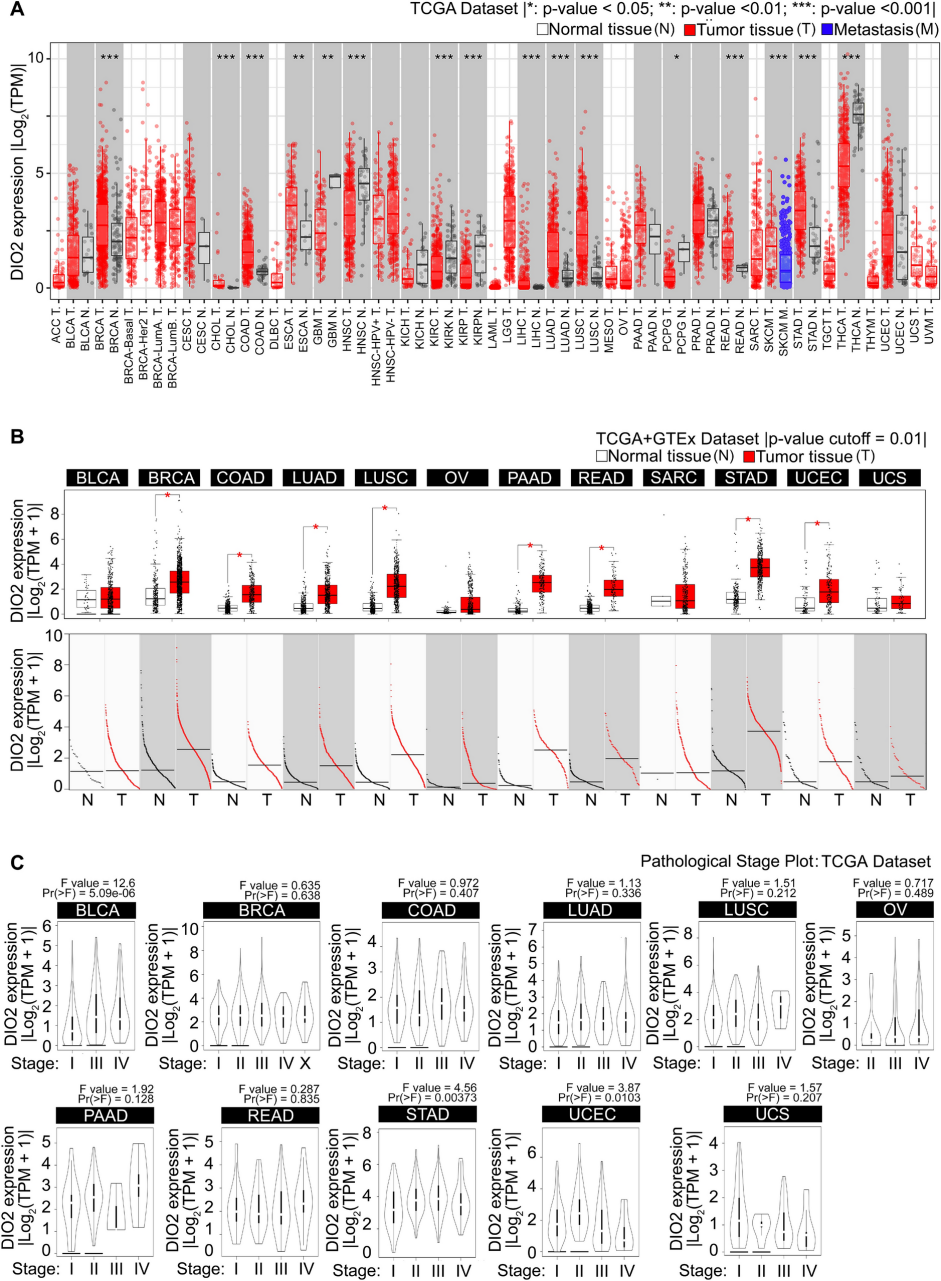
DIO2 gene expression in different tumors and pathological stages. **A** Differential DIO2 gene expression between tumor and adjacent normal tissues across all TCGA cancer types through the Gene_DE module of TIMER2.0 web server (http://timer.comp-genomics.org/timer/). DIO2 levels, expressed as|Log2(TPMs+1)|, are showed using box plots. **B** For the cancer types of BLCA, BRCA, COAD, LUAD, LUSC, OV, PAAD, READ, SARC, STAD, UCEC and UCS within the TCGA project, the corresponding normal tissues of the GTEx Database were included as controls. DIO2 levels, expressed as|Log2(TPMs+1)|, were analyzed through the DIY expression module of GEPIA tool (http://gepia.cancer-pku.cn) and showed as box plot. **C** Based on the TCGA Dataset, the expression levels of the DIO2 gene were analyzed by the main pathological stages through the Pathological Stage Plot module of GEPIA2 (http://gepia2.cancer-pku.cn). Statistical analyses were computed by the Wilcoxon test. * p-value < 0.05; ** p-value <0.01; *** p-value <0.001.

Unpaired analysis of DIO2 mRNA expression between tumor (T) and nontumor tissues (N) revealed that DIO2 expression levels in BLCA (Bladder Urothelial Carcinoma), BRCA (Breast invasive carcinoma), CESC (Cervical squamous cell carcinoma and endocervical adenocarcinoma), CHOL (Cholangiocarcinoma), COAD (Colon adenocarcinoma), ESCA (Esophageal carcinoma), LIHC (Liver Hepatocellular Carcinoma), LUAD (Lung adenocarcinoma), LUSC (Lung squamous cell carcinoma,), PAAD (Pancreatic adenocarcinoma), READ (Rectum adenocarcinoma), STAD (Stomach adenocarcinoma) and UCEC (Uterine Corpus Endometrial Carcinoma) are higher than the corresponding control tissues (for all p-value < 0.001) (Fig. 1A and Table 1). Given the lack of noncancer samples in some analysis, after including the normal tissue of the GTEx Dataset as controls, we further evaluated the DIO2 expression difference between tumor and normal tissues of OV (Ovarian serous cystadenocarcinoma), SARC (Sarcoma), STAD (Stomach adenocarcinoma) and UCS (Uterine Carcinosarcoma) (Fig. 1B and Table 2, all p-value < 0.01). However, the combined results of the TCGA + GTEx Datasets showed a significant higher expression of DIO2 in STAD, but there was no significant difference for the other three tumors, such as OV, SARC and UCS. Furthermore, we used the “Pathological Stage Plot” module of GEPIA2 to analyze the correlation between DIO2 expression and the patient’s pathological stages in different cancer types, and we found that the expression of DIO2 was significantly correlated with the pathological stages of BLCA, BRCA, COAD, LUAD, LUSC, OV, PAAD, READ, STAD, UCEC and UCS (Fig. 1C, all p-value < 0.05). Analysis of SARC Pathological Stage Plot was not possible due to lack of sufficient normal tissue samples. Overall, these data indicate that DIO2 is overexpressed in the majority of cancer types.

**Table 1:** DIO2 expression level between Tumor (T) and adjacent Normal (N) tissues or Metastasis (M) across all TCGA tumors through the Gene_DE module

TCGA acronym/tumor type	Number of Tumor (T), Normal (N) and Metastasis (M) samples	
ACC Adrenocortical carcinoma	Tumor sample (T)	n = 79
	Normal sample (N)	–
BLCA Bladder Urothelial Carcinoma	Tumor sample (T)	n = 408
	Normal sample (N)	n = 19
BRCA Breast invasive carcinoma	Tumor sample (T)	n = 1093
	Normal sample (N)	n = 112
BRCA-Basal Tumor	Tumor sample (T)	n = 190
BRCA-Her2 Tumor	Tumor sample (T)	n = 82
BRCA-LumA Tumor	Tumor sample (T)	n = 564
BRCA-LumB Tumor	Tumor sample (T)	n = 217
CESC Cervical squamous cell carcinoma and endocervical adenocarcinoma	Tumor sample (T)	n = 304
	Normal sample (N)	n = 3
CHOL Cholangiocarcinoma	Tumor sample (T)	n = 36
	Normal sample (N)	n = 9
COAD Colon adenocarcinoma	Tumor sample (T)	n = 457
	Normal sample (N)	n = 41
DLBC Diffuse large B cell lymphoma	Tumor sample (T)	n = 48
ESCA Esophageal carcinoma	Tumor sample (T)	n = 184
	Normal sample (N)	n = 11
GBM Glioblastoma multiforme	Tumor sample (T)	n = 153
	Normal sample (N)	n = 5
HNSC Head and Neck squamous cell carcinoma	Tumor sample (T)	n = 520
	Normal sample (N)	n = 44
HNSC-HPV + Tumor	Tumor sample (T)	n = 97
HNSC-HPV − Tumor	Tumor sample (T)	n = 421
KICH Kidney Chromophobe	Tumor sample (T)	n = 66
	Normal sample (N)	n = 25
KIRC Kidney renal clear cell carcinoma	Tumor sample (T)	n = 533
	Normal sample (N)	n = 72
KIRP Kidney renal papillary cell carcinoma	Tumor sample (T)	n = 290
	Normal sample (N)	n = 32
LAML Acute Myeloid Leukemia	Tumor sample (T)	n = 173
	Normal sample (N)	–
LGG Brain Lower Grade Glioma	Tumor sample (T)	n = 516
	Normal sample (N)	–
LIHC Liver hepatocellular carcinoma	Tumor sample (T)	n = 371
	Normal sample (N)	n = 50
LUAD Lung adenocarcinoma	Tumor sample (T)	n = 515
	Normal sample (N)	n = 59
LUSC Lung squamous cell carcinoma	Tumor sample (T)	n = 501
	Normal sample (N)	n = 51
MESO Mesothelioma	Tumor sample (T)	n = 87
	Normal sample (N)	–
OV Ovarian serous cystadenocarcinoma	Tumor sample (T)	n = 303
	Normal sample (N)	–
PAAD Pancreatic adenocarcinoma	Tumor sample (T)	n = 178
	Normal sample (N)	n = 4
PCPG Pheochromocytoma and Paraganglioma	Tumor sample (T)	n = 179
	Normal sample (N)	n = 3
PRAD Prostate adenocarcinoma	Tumor sample (T)	n = 497
	Normal sample (N)	n = 52
READ Rectum adenocarcinoma	Tumor sample (T)	n = 166
	Normal sample (N)	n = 10
SARC Sarcoma	Tumor sample (T)	n = 259
	Normal sample (N)	n = 103
SKCM Skin Cutaneous Melanoma	Metastasis (M)	n = 368
STAD Stomach adenocarcinoma	Tumor sample (T)	n = 415
	Normal sample (N)	n = 35
TGCT Testicular Germ Cell Tumors	Tumor sample (T)	n = 150
	Normal sample (N)	–
THYM Thymoma	Tumor sample (T)	n = 120
	Normal sample (N)	–
THCA Thyroid carcinoma	Tumor sample (T)	n = 501
	Normal sample (N)	n = 59
UCS Uterine Carcinosarcoma	Tumor sample (T)	n = 57
	Normal sample (N)	–
UCEC Uterine Corpus Endometrial Carcinoma	Tumor sample (T)	n = 545
	Normal sample (N)	n = 35
UVM Uveal Melanoma	Tumor sample (T)	n = 80
	Normal sample (N)	–

**Table 2:** DIO2 expression level between Tumor (T) and adjacent Normal (N) tissues or Metastasis (M) across all TCGA tumors through the “Expression Analysis-Box Plots” module of the GEPIA2

TCGA acronym/tumor type	Number of Tumor (T), Normal (N) and Metastasis (M) samples	
BLCA Bladder Urothelial Carcinoma	Tumor sample (T)	n = 404
	Normal sample (N)	n = 28
BRCA Breast invasive carcinoma	Tumor sample (T)	n = 1085
	Normal sample (N)	n = 291
COAD Colon adenocarcinoma	Tumor sample (T)	n = 275
	Normal sample (N)	n = 349
LUAD Lung adenocarcinoma	Tumor sample (T)	n = 483
	Normal sample (N)	n = 347
LUSC Lung squamous cell carcinoma	Tumor sample (T)	n = 486
	Normal sample (N)	n = 338
OV Ovarian serous cystadenocarcinoma	Tumor sample (T)	n = 426
	Normal sample (N)	n = 88
PAAD Pancreatic adenocarcinoma	Tumor sample (T)	n = 179
	Normal sample (N)	n = 171
READ Rectum adenocarcinoma	Tumor sample (T)	n = 92
	Normal sample (N)	n = 318
SARC Sarcoma	Tumor sample (T)	n = 262
	Normal sample (N)	n = 2
STAD Stomach adenocarcinoma	Tumor sample (T)	n = 408
	Normal sample (N)	n = 211
UCEC Uterine Corpus Endometrial Carcinoma	Tumor sample (T)	n = 174
	Normal sample (N)	n = 91
UCS Uterine Carcinosarcoma	Tumor sample (T)	n = 57
	Normal sample (N)	n = 78

### Survival analysis and prognostic value of DIO2 in pan-cancer

**Fig. 2:**
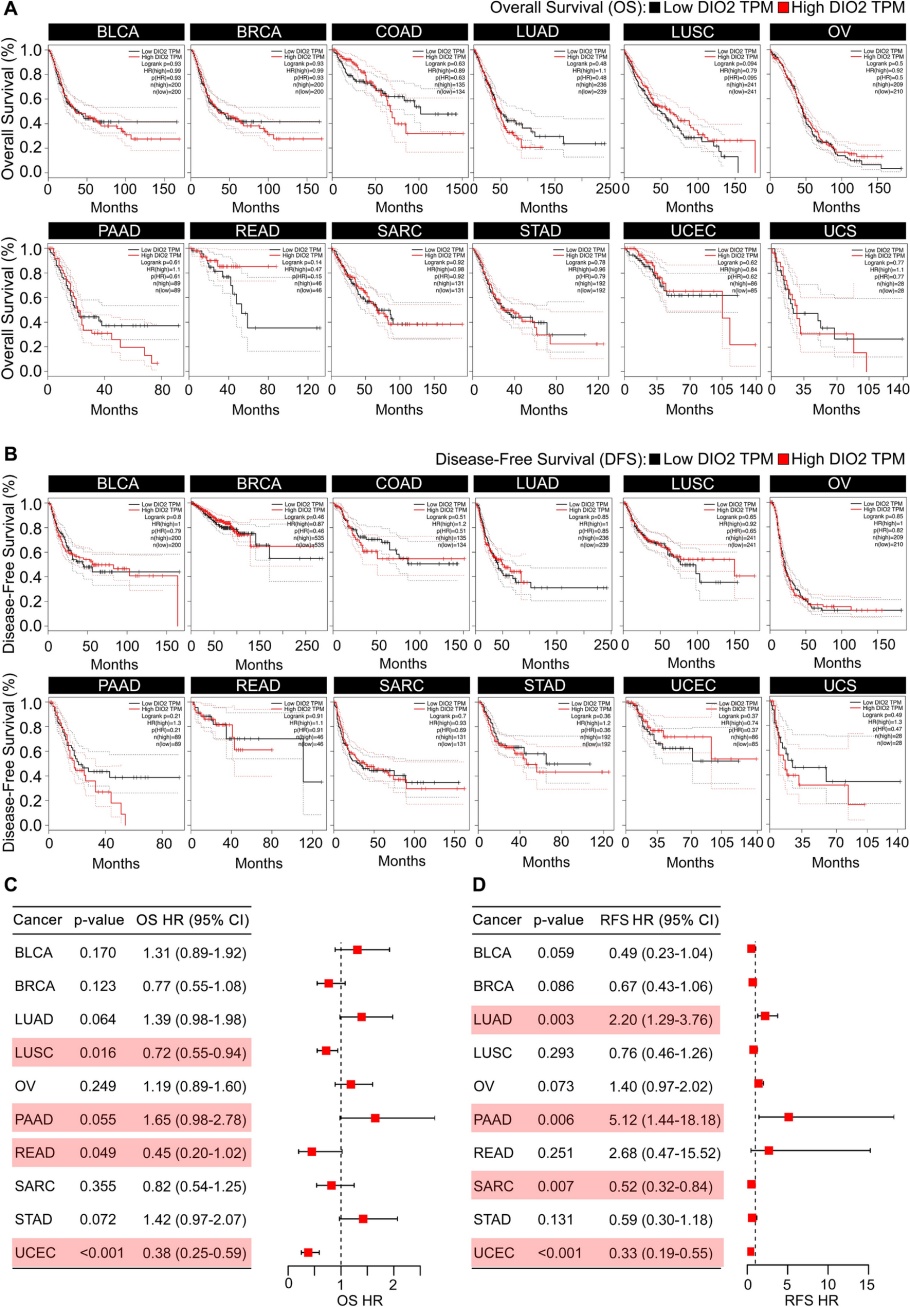
Correlation between DIO2 gene expression and patient’s prognosis in pan-cancer. **A** Overall Survival (OS) and **B** Disease-Free Survival (DFS) analyses of different cancer types within the TCGA Dataset by DIO2 gene expression were performed using the Survival Plot module of GEPIA2 tool. Kaplan-Meier curves for patient’s OS and DFS classified by low and high DIO2 gene expression levels in BLCA, BRCA, COAD, LUAD, LUSC, OV, PAAD, READ, SARC, STAD, UCEC and UCS. **C**, **D** Forest plots showed the correlation between DIO2 gene expression and OS (**C**) and Relapse-Free Survival (RFS) in different cancer types

To evaluate the prognostic assessment value of DIO2 in pan-cancer, we carried out Kaplan–Meier (KM) analysis and the Cox proportional hazards model. We divided the cancer cases into low- and high-DIO2 expression groups according to the DIO2 expression levels and investigated the correlation of DIO2 expression with the prognosis of patients with different tumors. Kaplan–Meier (KM) curves exhibited that patients with high DIO2 expression had worse Overall Survival (OS) than those with low DIO2 levels in BLCA, BRCA, COAD, LUAD, PAAD, SARC, STAD, UCEC and UCS within the TCGA Dataset. Conversely, high DIO2 expression was not linked to poor prognosis of OS for LUSC, OV and READ (Fig. 2A). Moreover, patients with high DIO2 expression had shortened Disease-Free Survival (DFS) interval in BLCA, LUAD, PAAD, READ, SARC, STAD and UCS compared to patients with low DIO2 expression. On the contrary, patients with low DIO2 expression had poor DFS in BRCA, COAD, LUSC and UCEC (Fig. 2B). Furthermore, Cox regression showed that a high DIO2 level was a risk factor in PAAD (OS HR = 1.65), while it served as a protective factor in LUSC (OS HR = 0.72, p-value = 0.016), READ (OS HR = 0.45) and UCEC (OS HR = 0.38) (Fig. 2C). For Relapse-Free Survival (RFS), Cox regression showed that a high DIO2 level was a risk factor for LUAD (RFS HR = 2.20) and PAAD (RFS HR = 5.12), while it served as a protective factor for SARC (RFS HR = 0.52) and UCEC (RFS HR = 0.52) (Fig. 2D).

### Genetic alteration of DIO2 in pan-cancer

To explore the mutational landscape of DIO2 in pan-cancer, we used cBioPortal online platform, that is a wide-ranging tool to query the genetic alteration frequency, mutation types and CNA (Copy Number Alteration) of a gene across all TCGA tumors. Although mutations of DIO2 genes have been reported rarely, the cBioPortal platform enabled to identify all the mutations for the DIO2 gene that have been annotated.

**Fig. 3:**
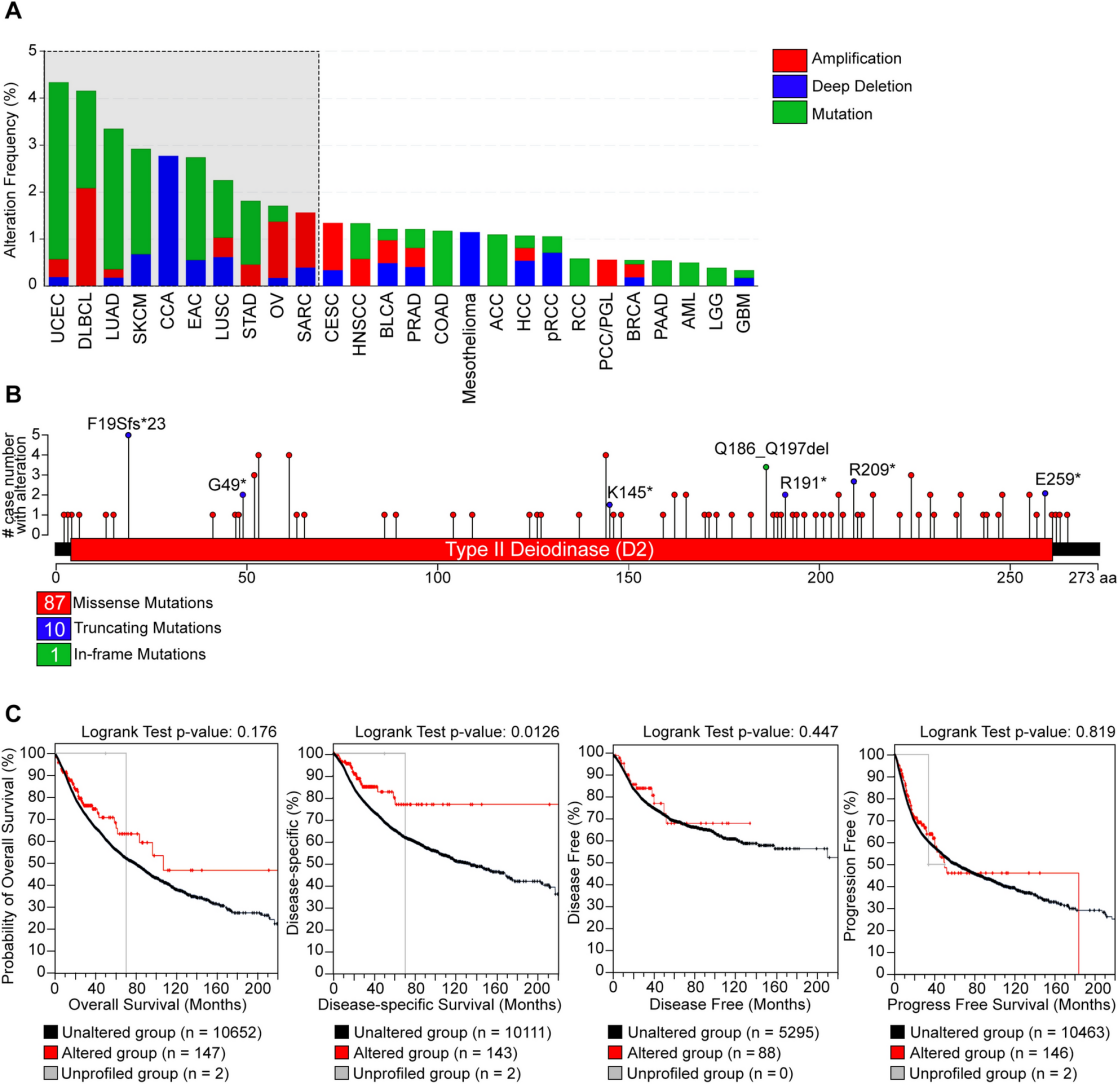
The genetic alterations of DIO2 in pan-cancer. **A** Genetic mutations analysis of DIO2 in pan-cancer was conducted by cBioPortal online platform (https://www.cbioportal.org) based on TCGA Dataset. **B** Mutation types, number and sites of DIO2 across protein domains. **C** Correlation between DIO2 mutation status and Overall Survival, Disease-specific Survival, Disease-Free Survival and Progress Free Survival of cancer patients are showed as individual Kaplan-Meier curves

Mutations with an alteration frequency below the 1.5% are considered not significantly associated with tumor progression for the modest number of events identified [39, 40], while when the frequency is above 1.5% the mutations are more significant and are indicated in the grey box of Fig. 3A. However, even with an alteration frequency lower than 1.5%, also other cancer types showed a general genomic alteration of DIO2: these tumors are reported in Fig. 3A, out of the gray box. The types, sites, and case number of the DIO2 genetic alteration are further shown in Fig. 3B and Table 3. Missense mutation of DIO2 was the main type of genetic alteration. As shown in Fig. 3B, a F19Sfs*23 alteration was detected in ACC, COAD, STAD and UCEC, which was able to induce a frame-shift mutation of the DIO2 gene, translation from F (Phenylalanine) to S (Serine) at the 19 sites of DIO2 protein. Furthermore, specific nonsense mutations were profiled in different types of cancer. This analysis did not show any increase in the alteration frequency in cancer compared to healthy tissue. However, importantly, when we analyzed the relevance of the mutations in DIO2 for the cancer prognosis, we observed an inverse correlation. Indeed, we used the “TCGA Pan-Cancer Atlas Studies” of cBioPortal online platform to explore the association between genetic alteration of DIO2 and the survival prognosis of different types of cancers. The data shown in Fig. 3C indicate that patients with altered DIO2 have a survival advantage in Overall Survival (OS) (p-value = 0.176), Disease-Specific Survival (p-value = 0.0126), Disease Free Survival (p-value = 0.447) and Progression Free Survival (p-value = 0.819) compared with cases without DIO2 alteration. Thus, this analysis suggests that DIO2 loss of function can confer an advantage for patient’s survival.

**Table 3:** Genetic alterations of DIO2 through the TCGA PanCancer Atlas: a summary of the 98 mutations annotated in cBioPortal online platform

Stydy of origin	Sample ID	Protein change	Mutation type	Copy	Allele frequency
Adrenocortical Carcinoma	TCGA-PK-A5HB-01	F19Sfs*23	FS del	Diploid	0.15
Brain Lower Grade Glioma	TCGA-DB-A4XB-01	*Q236E*	Missense	Diploid	0.48
Brain Lower Grade Glioma	TCGA-DU-6392–01	*A194T*	Missense	Diploid	0.34
Brain Lower Grade Glioma	TCGA-DU-6392–01	*V159I*	Missense	Diploid	0.28
Ovarian Serous Cystadenocarcinoma	TCGA-13–0885-01	*Q186_Q197del*	IF del	ShallowDel	0.21
Ovarian Serous Cystadenocarcinoma	TCGA-31–1950-01	*A226D*	Missense	ShallowDel	0.07
Glioblastoma Multiforme	TCGA-15–1446-01	*V210F*	Missense	Diploid	0.04
Diffuse Large B-Cell Lymphoma	TCGA-FA-A4XK-01	*R52W*	Missense	Diploid	0.45
Lung Squamous Cell Carcinoma	TCGA-56-A62T-01	*G2D*	Missense	Gain	0.28
Lung Squamous Cell Carcinoma	TCGA-33–4586-01	*R52L*	Missense	ShallowDel	0.55
Lung Squamous Cell Carcinoma	TCGA-34–7107-01	*R229C*	Missense	ShallowDel	0.31
Lung Squamous Cell Carcinoma	TCGA-33-AASB-01	*G243V*	Missense	Diploid	0.20
Lung Squamous Cell Carcinoma	TCGA-77–8143-01	*S263I*	Missense	Gain	0.58
Lung Squamous Cell Carcinoma	TCGA-77–8144-01	*V126F*	Missense	Diploid	0.30
Bladder Urothelial Carcinoma	TCGA-DK-A6AW-01	*R255W*	Missense	Diploid	0.39
Acute Myeloid Leukemia	TCGA-AB-2924–03	*A190T*	Missense	Diploid	
Liver Hepatocellular Carcinoma	TCGA-DD-AAW0-01	*P205T*	Missense	Diploid	0.21
Prostate Adenocarcinoma	TCGA-XK-AAIW-01	*R53H*	Missense	Diploid	0.53
Prostate Adenocarcinoma	TCGA-KK-A59V-01	*G49R*	Missense	Diploid	0.19
Kidney Renal Clear Cell Carcinoma	TCGA-AK-3456–01	*R229C*	Missense	Diploid	0.18
Kidney Renal Clear Cell Carcinoma	TCGA-CW-5591–01	*G224W*	Missense	Diploid	0.24
Kidney Renal Clear Cell Carcinoma	TCGA-A3-3319–01	*N261Y*	Missense	ShallowDel	0.53
Uterine Corpus Endometrial Carcinoma	TCGA-DI-A0WH-01	F19Sfs*23	FS del	Diploid	0.23
Uterine Corpus Endometrial Carcinoma	TCGA-EY-A215-01	F19Sfs*23	FS del	Diploid	0.34
Uterine Corpus Endometrial Carcinoma	TCGA-FI-A2D0-01	*R61H*	Missense	Diploid	0.37
Uterine Corpus Endometrial Carcinoma	TCGA-AP-A056-01	*R53H*	Missense	Diploid	0.36
Uterine Corpus Endometrial Carcinoma	TCGA-BK-A26L-01	*R61L*	Missense	Gain	0.14
Uterine Corpus Endometrial Carcinoma	TCGA-EO-A3AZ-01	*R255W*	Missense	Diploid	0.18
Uterine Corpus Endometrial Carcinoma	TCGA-A5-A0G1-01	R209*	Nonsense	Diploid	0.21
Uterine Corpus Endometrial Carcinoma	TCGA-A5-A0G1-01	*R144C*	Missense	Diploid	0.19
Uterine Corpus Endometrial Carcinoma	TCGA-A5-A0G1-01	*V89L*	Missense	Diploid	0.16
Uterine Corpus Endometrial Carcinoma	TCGA-A5-A1OF-01	*K244N*	Missense	Diploid	0.44
Uterine Corpus Endometrial Carcinoma	TCGA-A5-A2K5-01	*V6L*	Missense	Diploid	0.35
Uterine Corpus Endometrial Carcinoma	TCGA-AJ-A3EL-01	*K237N*	Missense	Diploid	0.22
Uterine Corpus Endometrial Carcinoma	TCGA-AP-A1DK-01	*R144H*	Missense	Diploid	0.23
Uterine Corpus Endometrial Carcinoma	TCGA-AX-A2HG-01	*R144H*	Missense	Diploid	0.30
Uterine Corpus Endometrial Carcinoma	TCGA-AP-A1DV-01	*E104D*	Missense	Diploid	0.38
Uterine Corpus Endometrial Carcinoma	TCGA-AX-A0J0-01	*R265I*	Missense	Diploid	0.46
Uterine Corpus Endometrial Carcinoma	TCGA-AX-A0J0-01	*L199R*	Missense	Diploid	0.59
Uterine Corpus Endometrial Carcinoma	TCGA-B5-A11E-01	*V230A*	Missense	Diploid	0.43
Uterine Corpus Endometrial Carcinoma	TCGA-BS-A0UF-01	*F247L*	Missense	Diploid	0.37
Uterine Corpus Endometrial Carcinoma	TCGA-D1-A103-01	*Q196H*	Missense	Diploid	0.25
Uterine Corpus Endometrial Carcinoma	TCGA-D1-A163-01	*A193T*	Missense	Diploid	0.39
Uterine Corpus Endometrial Carcinoma	TCGA-DI-A1BU-01	*I3T*	Missense	Diploid	0.36
Uterine Corpus Endometrial Carcinoma	TCGA-E6-A1LX-01	*Q13L*	Missense	Diploid	0.16
Uterine Corpus Endometrial Carcinoma	TCGA-FI-A2D0-01	*S177Y*	Missense	Diploid	0.37
Uterine Corpus Endometrial Carcinoma	TCGA-FI-A2D0-01	*R53C*	Missense	Diploid	0.32
Lung Adenocarcinoma	TCGA-17-Z015-01	*R48H*	Missense		0.44
Lung Adenocarcinoma	TCGA-05–4382-01	*T47N*	Missense	Diploid	0.36
Lung Adenocarcinoma	TCGA-55–8094-01	*R53P*	Missense	ShallowDel	0.85
Lung Adenocarcinoma	TCGA-86–8073-01	*L41F*	Missense	Diploid	0.12
Lung Adenocarcinoma	TCGA-97-A4M7-01	*R61L*	Missense	Diploid	0.13
Lung Adenocarcinoma	TCGA-17-Z031-01	*V211E*	Missense		0.43
Lung Adenocarcinoma	TCGA-44–2656-01	*A171E*	Missense	Diploid	0.21
Lung Adenocarcinoma	TCGA-44–3918-01	*T137K*	Missense	Diploid	0.15
Lung Adenocarcinoma	TCGA-44–6776-01	*L4I*	Missense	Diploid	0.32
Lung Adenocarcinoma	TCGA-55–7913-01	K145*	Nonsense	Diploid	0.27
Lung Adenocarcinoma	TCGA-55–8087-01	*D162G*	Missense	Diploid	0.08
Lung Adenocarcinoma	TCGA-55–8506-01	*Q188L*	Missense	Gain	0.40
Lung Adenocarcinoma	TCGA-73–4670-01	*R144S*	Missense	Diploid	0.38
Lung Adenocarcinoma	TCGA-78–7539-01	E259*	Nonsense	Diploid	0.62
Lung Adenocarcinoma	TCGA-86–8585-01	*D162Y*	Missense	Gain	0.36
Lung Adenocarcinoma	TCGA-93–8067-01	*V182L*	Missense	Gain	0.22
Lung Adenocarcinoma	TCGA-MP-A4TC-01	*R201L*	Missense	ShallowDel	0.22
Esophageal Adenocarcinoma	TCGA-LN-A49K-01	*L15M*	Missense	Gain	0.27
Esophageal Adenocarcinoma	TCGA-2H-A9GL-01	*I221R*	Missense	Gain	0.54
Esophageal Adenocarcinoma	TCGA-L5-A8NR-01	*L124P*	Missense	Diploid	0.56
Esophageal Adenocarcinoma	TCGA-LN-A49R-01	*L146M*	Missense	Diploid	0.52
Skin Cutaneous Melanoma	TCGA-D3-A5GN-06	*S248F*	Missense	ShallowDel	0.34
Skin Cutaneous Melanoma	TCGA-DA-A1HY-06	*S248F*	Missense	ShallowDel	0.52
Skin Cutaneous Melanoma	TCGA-EE-A2GM-06	*R191Q*	Missense	Gain	0.55
Skin Cutaneous Melanoma	TCGA-EB-A5SG-06	*G109R*	Missense	Diploid	0.20
Skin Cutaneous Melanoma	TCGA-EE-A2MJ-06	*P206L*	Missense	Gain	0.17
Skin Cutaneous Melanoma	TCGA-D3-A3CE-06	*G224W*	Missense	ShallowDel	0.15
Skin Cutaneous Melanoma	TCGA-D3-A8GI-06	*E189K*	Missense	ShallowDel	0.20
Skin Cutaneous Melanoma	TCGA-DA-A1I1-06	*H165N*	Missense	ShallowDel	0.08
Skin Cutaneous Melanoma	TCGA-EE-A29E-06	G49*	Nonsense	Diploid	0.11
Skin Cutaneous Melanoma	TCGA-EE-A2GK-06	*F262L*	Missense	Diploid	0.27
Stomach Adenocarcinoma	TCGA-HU-A4H8-01	F19Sfs*23	FS del	Diploid	0.23
Stomach Adenocarcinoma	TCGA-BR-8676–01	*V63I*	Missense	Diploid	0.42
Stomach Adenocarcinoma	TCGA-BR-6452–01	*R52W*	Missense	Diploid	0.13
Stomach Adenocarcinoma	TCGA-BR-7707–01	*G224R*	Missense	Diploid	0.28
Stomach Adenocarcinoma	TCGA-BR-8297–01	*K237T*	Missense	Diploid	0.08
Stomach Adenocarcinoma	TCGA-BR-8363–01	*N127T*	Missense	Diploid	0.10
Breast Invasive Carcinoma	TCGA-E2-A15R-01	*E148K*	Missense	Diploid	0.41
Pancreatic Adenocarcinoma	TCGA-IB-7651–01	*R214H*	Missense	Diploid	0.16
Colorectal Adenocarcinoma	TCGA-A6-2686–01	F19Sfs*23	FS del	Gain	0.18
Colorectal Adenocarcinoma	TCGA-AG-3909–01	*R214C*	Missense	Diploid	0.13
Colorectal Adenocarcinoma	TCGA-F5-6814–01	*R61C*	Missense	Diploid	0.57
Colorectal Adenocarcinoma	TCGA-AA-A02R-01	*P205L*	Missense	Diploid	0.32
Colorectal Adenocarcinoma	TCGA-AD-6889–01	*P173L*	Missense	Diploid	0.50
Colorectal Adenocarcinoma	TCGA-AF-A56K-01	*W257L*	Missense	Diploid	0.15
Colorectal Adenocarcinoma	TCGA-EI-6917–01	R191*	Nonsense	Diploid	0.23
Kidney Renal Papillary Cell Carcinoma	TCGA-KV-A6GE-01	*S203T*	Missense	Diploid	0.05
Head and Neck Squamous Cell Carcinoma	TCGA-CN-5369–01	*K65N*	Missense	Diploid	0.09
Head and Neck Squamous Cell Carcinoma	TCGA-CN-A497-01	*W170C*	Missense	ShallowDel	0.39
Head and Neck Squamous Cell Carcinoma	TCGA-CQ-7063–01	*H165Y*	Missense	Diploid	0.22
Head and Neck Squamous Cell Carcinoma	TCGA-UF-A71D-01	*V86M*	Missense	ShallowDel	0.24

### Analysis of DIO2 promoter methylation level in pan‑cancer

**Fig. 4:**
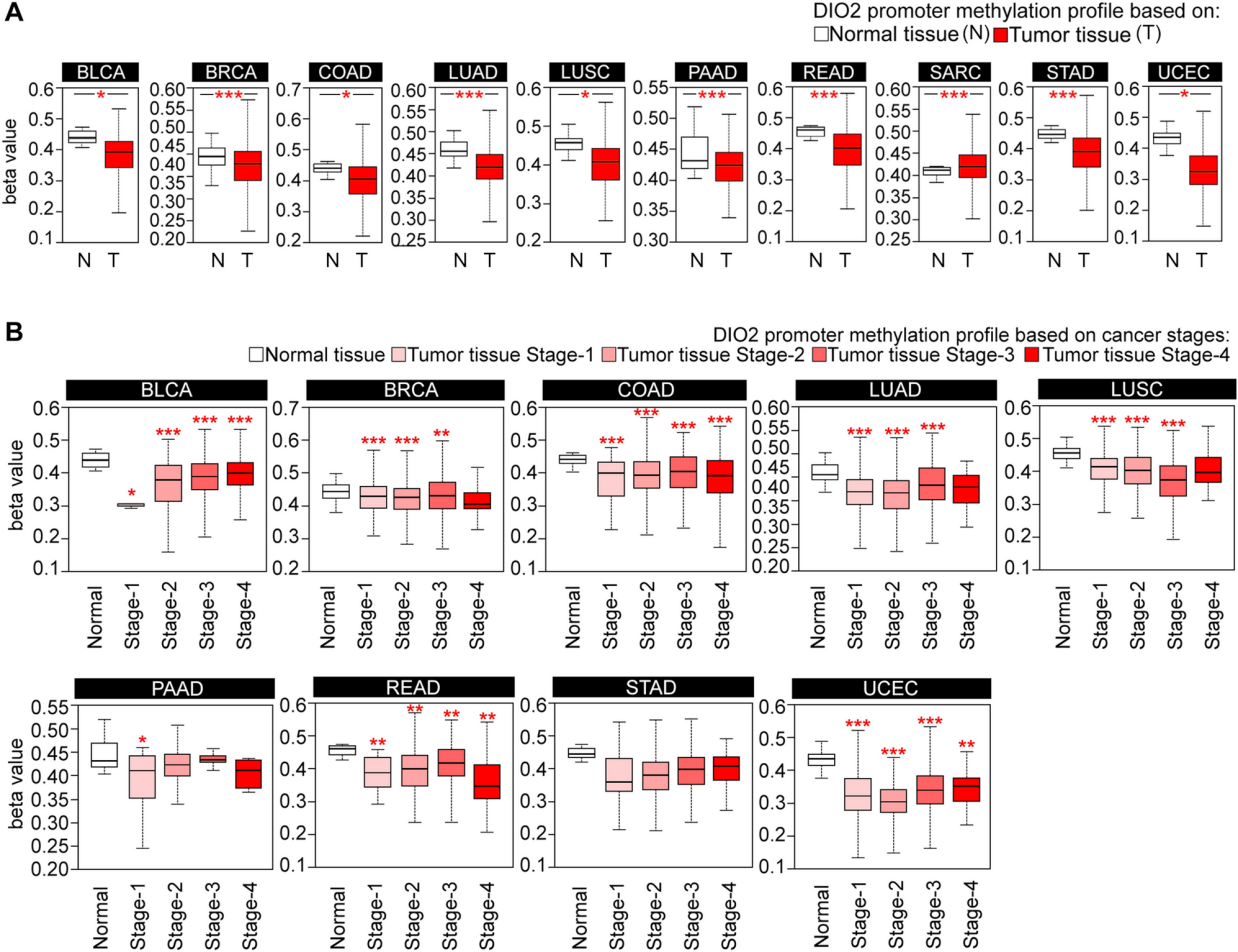
Analysis of DIO2 promoter methylation profile in pan‑cancer. **A** DIO2 promoter methylation levels between tumor and adjacent normal tissues across all TCGA cancer types through the UALCAN Database (https://ualcan.path.uab.edu). **B** DIO2 promoter methylation profile based on the individual cancer stages in BLCA, BRCA, COAD, LUAD, LUSC, PAAD, READ, STAD and UCEC. Statistical analyses were computed by the Wilcoxon test. * p-value < 0.05; ** p-value <0.01; *** p-value <0.001

To investigate the potential association between DIO2 promoter methylation and the pathogenesis of different cancer types in the TCGA Dataset, we carried out a DIO2 promoter methylation analysis using UALCAN Database. We compared the DIO2 promoter methylation level between cancer and normal tissues, and we found a significant negative correlation of DIO2 methylation status and the respective gene expression at multiple probes of the promoter region. In detail, we observed that the DIO2 promoter methylation level was significantly reduced in all the cancer types analyzed (Fig. 4A and Table 4). Similarly, we analyzed the DIO2 promoter methylation level also in individual cancer stages and confirmed this negative correlation between the methylation status and the respective gene expression (Fig. 4B and Table 5).

**Table 4:** DIO2 promoter methylation profile between Tumor (T) and adjacent Normal (N) tissues across all TCGA tumors through the UALCAN Database

(a) Promoter methylation level of DIO2 in BLCA					
TCGA samples	Parameters				
	Low	q1	Median	q3	High
Normal (n = 21)	0.406	0.423	0.439	0.46	0.472
Tumor (n = 418)	0.195	0.344	0.392	0.426	0.533

(b) Promoter methylation level of DIO2 in BRCA					
TCGA samples	Parameters				
	Low	q1	Median	q3	High
Normal (n = 97)	0.379	0.426	0.445	0.464	0.498
Tumor (n = 793)	0.276	0.391	0.428	0.456	0.573

(c) Promoter methylation level of DIO2 in COAD					
TCGA samples	Parameters				
	Low	q1	Median	q3	High
Normal (n = 37)	0.403	0.431	0.441	0.454	0.462
Tumor (n = 313)	0.221	0.358	0.405	0.444	0.584

(d) Promoter methylation level of DIO2 in LUAD					
TCGA samples	Parameters				
	Low	q1	Median	q3	High
Normal (n = 32)	0.418	0.445	0.456	0.477	0.502
Tumor (n = 473)	0.296	0.393	0.42	0.448	0.548

(e) Promoter methylation level of DIO2 in LUSC					
TCGA samples	Parameters				
	Low	q1	Median	q3	High
Normal (n = 42)	0.411	0.44	0.457	0.468	0.504
Tumor (n = 370)	0.255	0.362	0.407	0.441	0.561

(f) Promoter methylation level of DIO2 in PAAD					
TCGA samples	Parameters				
	Low	q1	Median	q3	High
Normal (n = 10)	0.403	0.419	0.431	0.469	0.519
Tumor (n = 184)	0.339	0.399	0.424	0.444	0.507

(g) Promoter methylation level of DIO2 in READ					
TCGA samples	Parameters				
	Low	q1	Median	q3	High
Normal	0.426	0.443	0.461	0.47	0.474
(n = 7)					
Tumor	0.207	0.347	0.403	0.447	0.578
(n = 98)					

(h) Promoter methylation level of DIO2 in SARC					
TCGA samples	Parameters				
	Low	q1	Median	q3	High
Normal (n = 4)	0.385	0.401	0.412	0.418	0.419
Tumor (n = 261)	0.302	0.396	0.42	0.445	0.539

(i) Promoter methylation level of DIO2 in STAD					
TCGA samples	Parameters				
	Low	q1	Median	q3	High
Normal (n = 2)	0.42	0.434	0.447	0.461	0.474
Tumor (n = 395)	0.202	0.342	0.391	0.433	0.573

(j) Promoter methylation level of DIO2 in UCEC					
TCGA samples	Parameters				
	Low	q1	Median	q3	High
Normal (n = 46)	0.376	0.415	0.435	0.449	0.489
Tumor (n = 438)	0.149	0.285	0.325	0.375	0.52

(a) p-value Normal-vs-Tumor = 1.63E + 02

(b) p-value Normal-vs-Tumor = 2.22E + 07

(c) p-value Normal-vs-Tumor = 1.62E + 02

(d) p-value Normal-vs-Tumor = 1.50E + 06

(e) p-value Normal-vs-Tumor = 1.76E + 02

(f) p-value Normal-vs-Tumor = 1.91E + 05

(g) p-value Normal-vs-Tumor = 2.96E + 07

(h) p-value Normal-vs-Tumor = 6.00E + 05

(i) p-value Normal-vs-Tumor = 3.35E + 05

(j) p-value Normal-vs-Tumor = 1.62E + 02

**Table 5:** DIO2 promoter methylation profile between individual cancer stages of Tumor (T) and adjacent Normal (N) tissues across all TCGA tumors through the UALCAN Database

Promoter methylation level of DIO2 in BLCA							
TCGA samples	Parameters						
	Low	q1	Median	q3	High		
Normal (n = 21)	0.406	0.423	0.439	0.46	0.472		
Stage1 (n = 4)	0.293	0.3	0.307	0.307	0.307	p-value Normal-vs Stage1	2.98E+04
Stage2 (n = 131)	0.16	0.315	0.381	0.424	0.503	p-value Normal-vs Stage 2	1.68E+02
Stage3 (n = 143)	0.206	0.349	0.391	0.429	0.532	p-value Normal-vs Stage 3	3.24E+03
Stage4 (n = 138)	0.257	0.364	0.403	0.432	0.533	p-value Normal-vs Stage 4	3.31E+06

Promoter methylation level of DIO2 in BRCA							
TCGA samples	Parameters						
	Low	q1	Median	q3	High		
Normal (n = 97)	0.379	0.426	0.445	0.464	0.498		
Stage1 (n = 127)	0.308	0.394	0.43	0.458	0.569	p-value Normal-vs-Stage1	8.43E+09
Stage2 (n = 442)	0.283	0.39	0.427	0.453	0.568	p-value Normal-vs-Stage2	2.58E+08
Stage3 (n = 200)	0.269	0.393	0.431	0.47	0.598	p-value Normal-vs-Stage3	8.20E+03
Stage4 (n = 11)	0.328	0.392	0.406	0.44	0.517		

Promoter methylation level of DIO2 in COAD							
TCGA samples	Parameters						
	Low	q1	Median	q3	High		
Normal (n = 37)	0.403	0.431	0.441	0.454	0.462		
Stage1 (n = 50)	0.227	0.33	0.4	0.433	0.478	p-value Normal-vs-Stage1	2.18E+02
Stage2 (n = 122)	0.221	0.364	0.404	0.444	0.579	p-value Normal-vs-Stage2	3.60E+05
Stage3 (n = 88)	0.232	0.356	0.405	0.449	0.524	p-value Normal-vs-Stage3	1.16E+08
Stage4 (n = 41)	0.174	0.34	0.392	0.438	0.543	p-value Normal-vs-Stage4	5.63E+09

Promoter methylation level of DIO2 in LUAD							
TCGA samples	Parameters						
	Low	q1	Median	q3	High		
Normal (n = 32)	0.418	0.445	0.456	0.477	0.502		
Stage1 (n = 260)	0.298	0.392	0.418	0.444	0.535	p-value Normal-vs-Stage1	2.70E+06
Stage2 (n = 115)	0.291	0.384	0.418	0.442	0.534	p-value Normal-vs-Stage2	1.66E+08
Stage3 (n = 73)	0.309	0.402	0.433	0.469	0.544	p-value Normal-vs-Stage3	6.60E+02
Stage4 (n = 20)	0.344	0.396	0.43	0.453	0.484		

Promoter methylation level of DIO2 in LUSC							
TCGA samples	Parameters						
	Low	q1	Median	q3	High		
Normal (n = 42)	0.411	0.44	0.457	0.468	0.504		
Stage1 (n = 172)	0.275	0.378	0.416	0.44	0.537	p-value Normal-vs-Stage1	9.78E+03
Stage2 (n = 135)	0.257	0.362	0.405	0.442	0.534	p-value Normal-vs-Stage2	4.79E+08
Stage3 (n = 56)	0.192	0.326	0.375	0.416	0.525	p-value Normal-vs-Stage3	8.33E+09
Stage4 (n = 4)	0.311	0.366	0.397	0.442	0.538		

Promoter methylation level of DIO2 in PAAD							
TCGA samples	Parameters						
	Low	q1	Median	q3	High		
Normal (n = 3)	0.403	0.419	0.431	0.469	0.519		
Stage1 (n = 21)	0.245	0.352	0.412	0.442	0.46	p-value Normal-vs-Stage1	2.86E+04
Stage2 (n = 145)	0.339	0.4	0.424	0.445	0.507		
Stage3 (n = 3)	0.411	0.429	0.435	0.442	0.458		
Stage4 (n = 4)	0.365	0.373	0.413	0.433	0.436		

Promoter methylation level of DIO2 in READ							
TCGA samples	Parameters						
	Low	q1	Median	q3	High		
Normal (n = 7)	0.426	0.443	0.461	0.47	0.474		
Stage1 (n = 11)	0.293	0.345	0.389	0.434	0.458	p-value Normal-vs-Stage1	3.24E+03
Stage2 (n = 29)	0.244	0.355	0.409	0.448	0.578	p-value Normal-vs-Stage2	2.96E+03
Stage3 (n = 35)	0.237	0.379	0.418	0.459	0.549	p-value Normal-vs-Stage3	2.80E+03
Stage4 (n = 13)	0.207	0.309	0.347	0.411	0.542	p-value Normal-vs-Stage4	2.06E+03

Promoter methylation level of DIO2 in STAD					
TCGA samples	Parameters				
	Low	q1	Median	q3	High
Normal (n = 2)	0.42	0.434	0.447	0.461	0.474
Stage1 (n = 52)	0.215	0.332	0.362	0.432	0.542
Stage2 (n = 125)	0.211	0.336	0.382	0.419	0.549
Stage3 (n = 174)	0.237	0.353	0.4	0.435	0.552
Stage4 (n = 33)	0.274	0.366	0.409	0.437	0.492

Promoter methylation level of DIO2 in UCEC							
TCGA samples	Parameters						
	Low	q1	Median	q3	High		
Normal (n = 46)	0.376	0.415	0.435	0.449	0.489	p-value Normal-vs-Primary	1.62E+02
Stage1 (n = 244)	0.134	0.28	0.324	0.374	0.522		
Stage2 (n = 43)	0.149	0.273	0.304	0.342	0.439		
Stage3 (n = 106)	0.163	0.298	0.342	0.383	0.532		
Stage4 (n = 24)	0.234	0.307	0.355	0.376	0.457		

### Immunogenomic analysis of DIO2 in pan-cancer

**Fig. 5:**
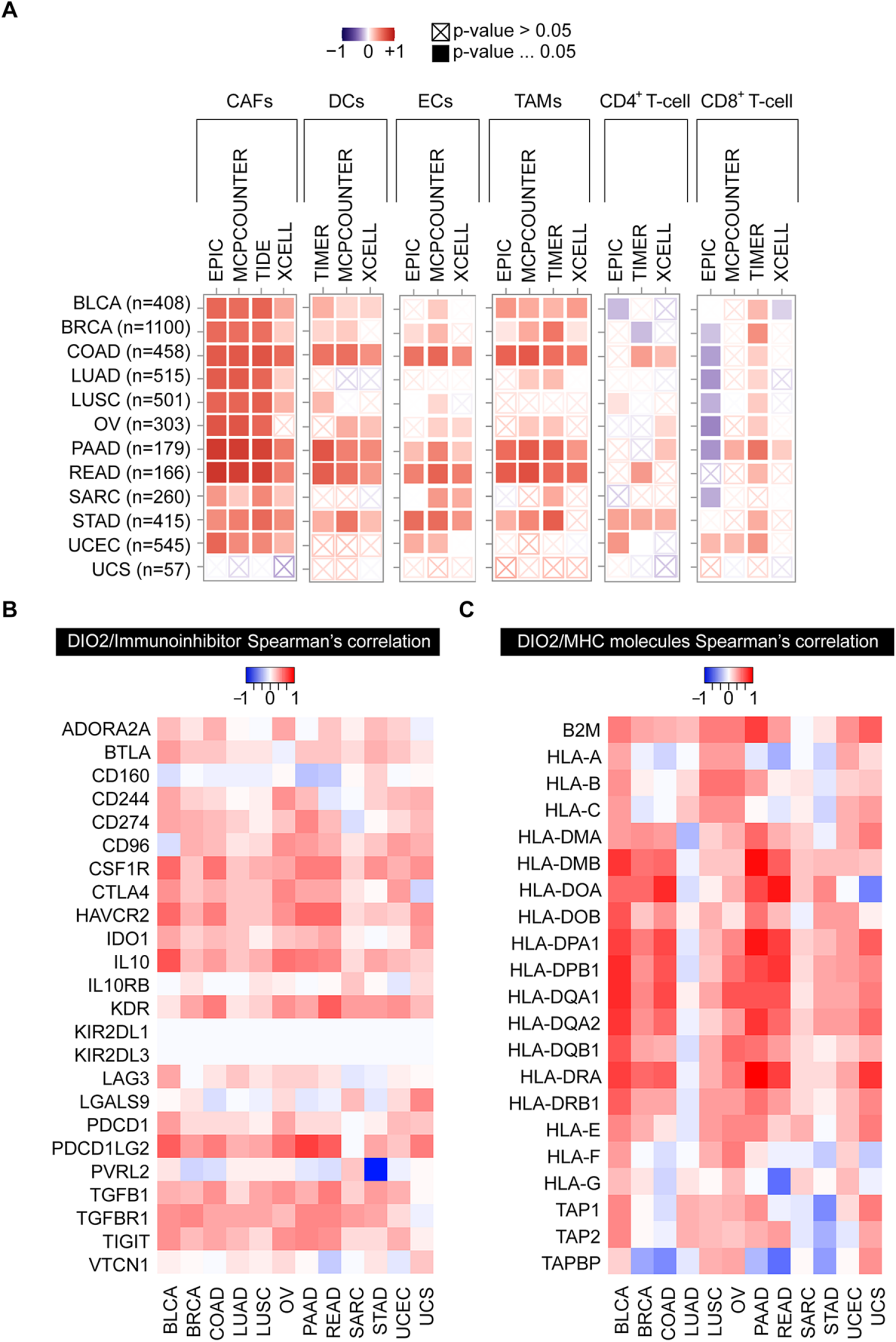
Correlation analysis between DIO2 expression and immune and endothelial cells infiltration in pan-cancer. **A** The potential correlation between the expression level of the DIO2 gene and the infiltration level of Cancer-Associated Fibroblasts (CAFs), Dendritic Cells (DCs), Endothelial Cells (ECs), Tumor-Associated Macrophages (TAMs) and of CD4+ and CD8+ T-cells was analyzed by different algorithms across all types of cancer in TCGA through the Immune-Gene module of the TIMER2.0 web server. Correlation analyses were computed by Spearman’s rank correlation test (Spearman correlation coefficient, R). Significant positive correlation (p-value < 0.05, R > 0). Significant negative correlation (p-value < 0.05, R < 0). Not significant (p-value > 0.05). **B**, **C** Correlations of DIO2 expression with the expression of Immunoinhibitors (**B**) and MHC molecules (**C**) in the TISIDB Database (http://cis.hku.hk/TISIDB/). Red and blue represent positive and negative correlations, respectively

We examined the potential relationship between DIO2 gene expression and the infiltration of immune and endothelial cells in different TCGA cancer types applying the TIMER2.0 approach with different algorithms, such as EPIC, MCPCOUNTER, XCELL, TIDE and TIMER. As shown in Fig. 5A, we found a positive correlation between DIO2 expression and the infiltration of Cancer-Associated Fibroblasts (CAFs) in all the cancer types analyzed, with the exception of UCS in which we identified a not-significant negative correlation (Supplemental Fig. S1, 2). In addition, DIO2 expression was positively correlated to Dendritic Cells (DCs) (Supplemental Fig. S3, 4), Endothelial Cells (ECs) (Supplemental Fig. S5, 6) and Tumor-Associated Macrophages (TAMs) (Supplemental Fig. S7, 8) in all the cancer types analyzed. Moreover, DIO2 expression was significantly related to the infiltration of CD4^+^ T-cell (Supplemental Fig. S9, 10) and CD8^+^ T-cell (Supplemental Fig. S11, 12) (i.e. COAD, LUSC, OV, PAAD, READ, STAD and UCEC). Furthermore, since tumor immunotherapy has been proven to be an effective novel therapeutic strategy against multiple types of cancers, we explored the possibility of using DIO2 as a novel target for tumor immunotherapy. Thus, we analyzed the relationship between the expression of DIO2 and several immunomodulatory genes and molecules by gene co-expression analyses in the TISIDB Database and we found that in most cancers DIO2 expression was significantly correlated with immunoinhibitory and immunostimulatory genes, as well as Major Histocompatibility Complex (MHC) molecules, chemokines, and chemokine receptors (Fig. 5B, C and Supplemental Fig. S13A-C). We found a positive correlation between DIO2 and NF-kB (that up-regulates DIO2 expression [41]) in pan-cancer analysis and in specific cancer types (Supplemental Fig. S14). The above findings proved that DIO2 is linked to immune and endothelial infiltration in pan-cancer and demonstrated that DIO2 expression is significantly correlated with multiple immune inhibitors, immune stimulators, and MHC molecules in multiple cancer types.

### Functional enrichment of DIO2‑related partners in pan-cancer

**Fig. 6:**
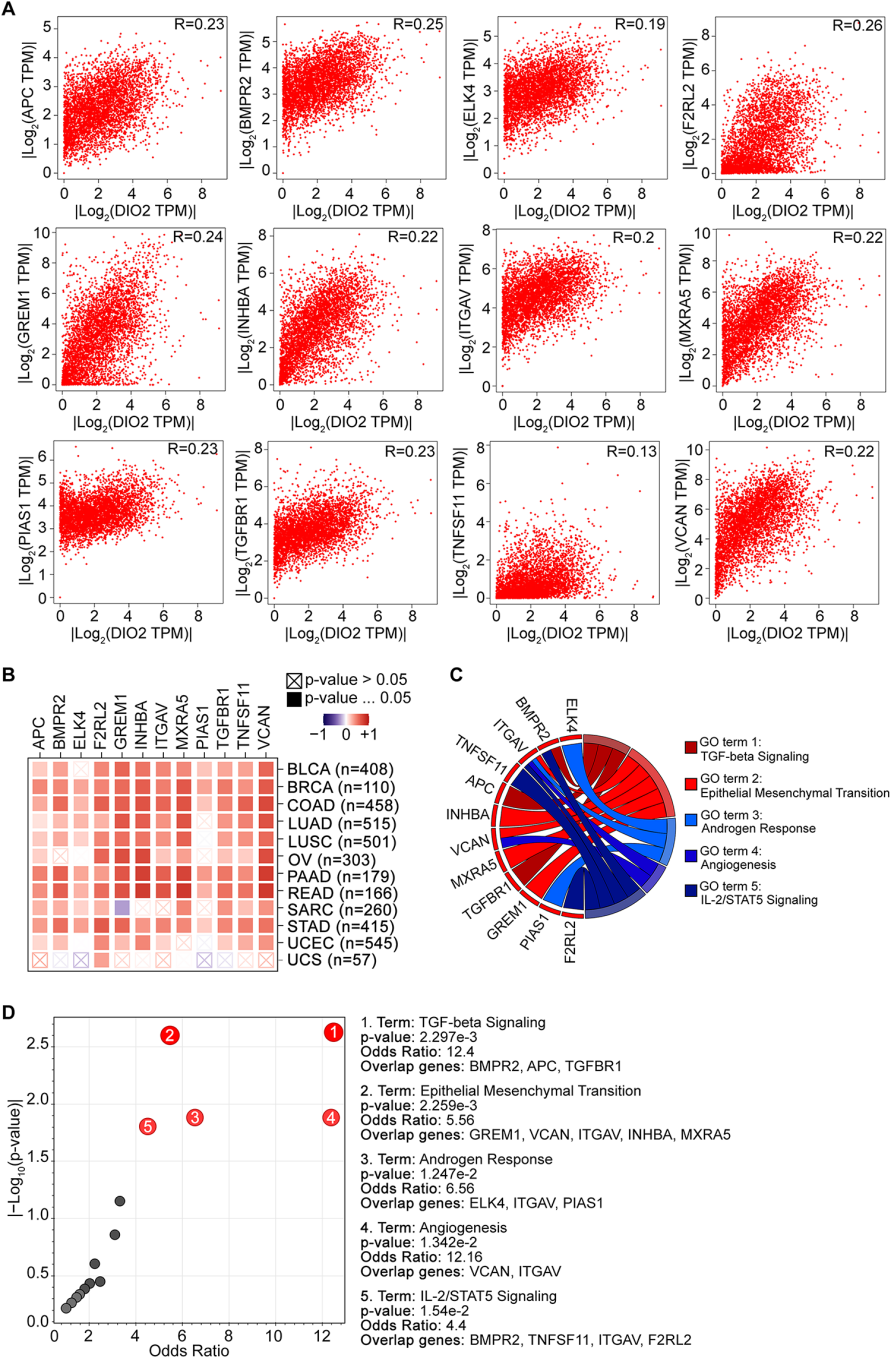
DIO2-related gene enrichment analysis. **A** Correlation analyses between twelve of the top 100 DIO2-correlated genes in TCGA project, including APC (R = 0.23), BMPR2 (R = 0.25), ELK4 (R = 0.19), F2RL2 (R = 0.26), GREM1 (R = 0.24), INHBA (R = 0.22), ITGAV (R = 0.2), MXRA5 (R = 0.22), PIAS1 (R = 0.23), TGFBR1 (R = 0.23), TNFSF11 (R = 0.13) and VCAN (R = 0.22) (all p-value < 0.001). Correlation analyses were analyzed by Similar Genes Detection tool of GEPIA2 and computed by Spearman’s rank correlation test (Spearman correlation coefficient, R). Significant positive correlation (p-value < 0.05, R > 0). **B** The corresponding correlation data in the detailed cancer types are displayed as heatmap. **C**, **D** Based on the DIO2-interacted genes, Gene Ontology (GO) enrichment analysis in Biological Processes (BP) was performed by using the Enrichr suite of gene set enrichment analysis tool (https://maayanlab.cloud/Enrichr/). GO enriched terms are visualized as GO chord (**C**) and Volcano plot (**D**) from the MSigDB_Hallmark_2020 gene set

To further investigate the molecular mechanism of the DIO2 gene in tumorigenesis, we attempted to screen out the targeting DIO2 expression-correlated genes for pathway enrichment analysis. We used the “Similar Genes Detection” tool of GEPIA2 to combine all tumor expression data of TCGA Dataset and obtained the top 100 genes that correlated with DIO2 expression in pan-cancer (Table 6). Among these, twelve genes displayed strong correlation with DIO2 in most cancer types. As shown in Fig. 6A, DIO2 was positively correlated with the expression of APC (Adenomatous Polyposis Coli, R = 0.23), BMPR2 (Bone Morphogenetic Protein Receptor type II, R = 0.25), ELK4 (ETS-like transcription factor 4, R = 0.19), F2RL2 (coagulation Factor II Receptor-Like 2, R = 0.26), GREM1 (Gremlin 1, R = 0.24), INHBA (Inhibin Subunit Beta A, R = 0.22), ITGAV (Integrin Subunit Alpha V, R = 0.2), MXRA5 (MatriX-Remodelling Associated proteins, R = 0.22), PIAS1 (Protein Inhibitor of Activated STAT 1, R = 0.23), TGFBR1 (Transforming Growth Factor-Beta Receptor type 1, R = 0.23), TNFSF11 (TNF Super-Family member 11, R = 0.13) and VCAN (Versican, R = 0.22) (all p-value < 0.001). The corresponding heatmap data also showed a positive correlation between DIO2 and the above twelve genes in the majority of detailed cancer types (Fig. 6B). Furthermore, the Gene Ontology (GO) enrichment analysis in Biological Processes (BP) indicated that these genes are directly or indirectly linked to TGF-beta signaling, Epithelial Mesenchymal Transition, Androgen Response, Angiogenesis, and IL-2/STAT5 signaling (Fig. 6C-D).

**Table 6:** Top 100 similar genes showing an analogous expression pattern with DIO2 gene in pan-cancer

Gene symbol	Gene ID	PCC
FBP2P1	ENSG00000237743.3	0.45
CTB-43I4.1	ENSG00000279743.1	0.44
RP11-184M15.1	ENSG00000248187.1	0.40
CEP128	ENSG00000100629.16	0.31
CA10	ENSG00000154975.13	0.31
C2CD4A	ENSG00000198535.5	0.29
RP11-1081L13.4	ENSG00000254966.1	0.27
DENND4C	ENSG00000137145.20	0.27
F2R	ENSG00000181104.6	0.27
F2RL2	ENSG00000164220.6	0.25
FOCAD	ENSG00000188352.12	0.25
KLHL9	ENSG00000198642.6	0.25
VPS53	ENSG00000141252.19	0.24
PNPO	ENSG00000108439.9	0.24
PPP3CA	ENSG00000138814.16	0.23
FOXP1	ENSG00000114861.18	0.23
RP11-244H3.4	ENSG00000271741.1	0.23
MPHOSPH6	ENSG00000135698.9	0.23
RP11-815J21.4	ENSG00000259367.1	0.23
MAP3K2	ENSG00000169967.16	0.23
SFMBT2	ENSG00000198879.11	0.23
ENTPD1	ENSG00000138185.16	0.23
SERHL2	ENSG00000183569.17	0.23
GREM1	ENSG00000166923.10	0.23
PIAS1	ENSG00000033800.13	0.23
CNOT6L	ENSG00000138767.12	0.22
ATRX	ENSG00000085224.20	0.22
TRIP11	ENSG00000100815.12	0.22
SLC30A7	ENSG00000162695.11	0.22
GUCY1A3	ENSG00000164116.16	0.22
KIAA1033	ENSG00000136051.13	0.22
TGFBR1	ENSG00000106799.12	0.22
ZMAT3	ENSG00000172667.10	0.22
FTX	ENSG00000230590.7	0.22
AC066694.1	ENSG00000281204.1	0.22
SEC24A	ENSG00000113615.12	0.22
KIAA0825	ENSG00000185261.13	0.22
MXRA5	ENSG00000101825.7	0.22
FEM1C	ENSG00000145780.7	0.22
RP11-588K22.2	ENSG00000260244.1	0.22
ANTXR1	ENSG00000169604.19	0.21
TMEM86A	ENSG00000151117.8	0.21
VCAN	ENSG00000038427.15	0.21
UBR1	ENSG00000159459.11	0.21
APC	ENSG00000134982.16	0.21
ADAMTS12	ENSG00000151388.10	0.21
MFAP3	ENSG00000037749.11	0.21
FTOP1	ENSG00000226491.1	0.21
TCF4	ENSG00000196628.13	0.21
RP11-449H3.3	ENSG00000275740.1	0.21
KMO	ENSG00000117009.11	0.21
PSMD5	ENSG00000095261.13	0.21
RP11-507B12.1	ENSG00000259675.1	0.21
ZNF460	ENSG00000197714.8	0.21
TCF12	ENSG00000140262.17	0.21
NCOA1	ENSG00000084676.15	0.21
INHBA	ENSG00000122641.9	0.21
ZBTB20	ENSG00000181722.15	0.20
ARID4A	ENSG00000032219.18	0.20
NFAT5	ENSG00000102908.20	0.20
PURA	ENSG00000185129.5	0.20
DPP8	ENSG00000074603.18	0.20
PRDM1	ENSG00000057657.14	0.20
STAM2	ENSG00000115145.9	0.20
TNFSF11	ENSG00000120659.14	0.20
SEC24B	ENSG00000138802.11	0.20
DDX6	ENSG00000110367.11	0.20
RP11-357N13.6	ENSG00000280392.1	0.20
BMP8A	ENSG00000183682.7	0.20
FAM160B1	ENSG00000151553.14	0.20
ANKRD12	ENSG00000101745.15	0.20
RNF11	ENSG00000123091.4	0.20
TMEM87B	ENSG00000153214.9	0.20
DTWD2	ENSG00000169570.9	0.20
KLRC4-KLRK1	ENSG00000255819.6	0.20
GTF2A1	ENSG00000165417.11	0.20
SLAIN2	ENSG00000109171.14	0.20
NFE2L1	ENSG00000082641.15	0.20
LINC01105	ENSG00000232044.6	0.20
TET2	ENSG00000168769.12	0.20
RANBP2	ENSG00000153201.15	0.20
TC2N	ENSG00000165929.12	0.20
IDE	ENSG00000119912.15	0.20
NEDD4	ENSG00000069869.15	0.20
AFF4	ENSG00000072364.12	0.20
ZBED6	ENSG00000257315.1	0.20
PEAK1	ENSG00000173517.10	0.20
INO80D	ENSG00000114933.15	0.20
NFIA	ENSG00000162599.15	0.20
SPTY2D1	ENSG00000179119.14	0.20
SP2	ENSG00000167182.12	0.20
GCC2	ENSG00000135968.19	0.20
GSN-AS1	ENSG00000235865.2	0.20
BMPR2	ENSG00000204217.12	0.20
ITGAV	ENSG00000138448.11	0.20
GPR137B	ENSG00000077585.13	0.19
ZNF562	ENSG00000171466.9	0.19
CCSER2	ENSG00000107771.15	0.19
ZNF292	ENSG00000188994.12	0.19
ELK4	ENSG00000158711.13	0.19

Pearson’s Correlation Coefficients (PCCs) are provided

## Discussion

Cancer is driven by genetic mutations, which in turn cause alterations of cellular pathways leading cancer cells to lose the normal constraints on cell growth, to modify the local microenvironment, to acquire invasive abilities, spread to other organs and finally, to evade the immune surveillance. The detection of genetic alterations is thus of critical need for precision treatment of cancer.

Thyroid Hormone (TH) signal is among the endocrine regulators of such a variety of cellular functions that when altered can worse the cancer risk. Although many studies have shown that the intracellular activation of TH can cause cancer invasiveness and spreading, our knowledge about the clinical relevance of such a process is still missing. Here, we describe the clino-pathological relevance of the DIO2 gene, encoding for the D2 protein, the TH activating enzyme, in a diversity of human cancers.

In our study DIO2 appears as an adverse prognostic factor in terms of OS in patients with different cancers. This observation is in line with the concept that TH activation via D2 increases the angiogenesis, rewrites the cancer cells metabolism, and fosters the Epithelial-to-Mesenchymal Transition (EMT) [13, 20]. While the DIO2 gene positively correlates with oncogenic features and worsen the cancer aggressiveness, to date no strict associations have been reported between the occurrence of the Thr/Ala DIO2 polymorphism and cancer frequency [42, 43].

One intriguing speculation can be raised on the possibility that the higher expression of DIO2 in cancer might be a compensatory change evoked by decreased T3 availability in cancer cells. However, it has been described that while intratumoral T3 levels are low in benign tumors (for instance in the papillomas of the skin), the intratumoral T3 raises in more invasive tumors along with the increase in D2 levels [20]. Therefore, this indicates that D2 elevation causes the TH activation and that D2 induction is a reason and not a consequence of a putative hypothyroidism in high grade tumors. Moreover, in the Squamous Cell Carcinoma (SCC), DIO2 genetic depletion attenuates the invasiveness and metastatic formation [20]. This proves that D2 is responsible for the evolution of tumors toward malignancy.

One of the major challenges in the tumor biology is the search for new prognostic and predictive biomarkers enabling to improve the tumoral outcome and to better select the specific anticancer treatment. A cancer biomarker can be generally defined as a tumoral feature that can be objectively measured and easily quantified as an indicator of tumoral formation and/or progression. In addition, if the biomarker has also a predictive value, it can provide information on the effects of a therapeutic intervention. Importantly, biomarkers can be target for therapy.

From this point of view, one of the most relevant finding of our study is that it identifies DIO2 as a potential cancer biomarker. This conclusion is based on different solid observations of our study: (1) DIO2 expression is an independent prognostic factor in many different solid tumors; (2) patients with high DIO2 expression have a lower survival compared to patients with lower DIO2 levels; (3) DIO2 expression positively correlates with immune and endothelial infiltration in cancer; (4) mutations in DIO2 gene ameliorate the free-disease survival.

In agreement with the concept that the methylation level in the promoter region of a tumor-associated gene can influence its function as oncogene or anti-oncogene [44–46], we found that the hypomethylation of DIO2 in cancer leads to the overexpression of the DIO2 gene and affects the occurrence and progression of tumors.

Finally, we predicted the KEGG (Kyoto Encyclopedia of Genes and Genomes) pathways associated with DIO2. DIO2 was involved in “TGF-beta signaling”, “Epithelial Mesenchymal Transition”, “Androgen Response”, “Angiogenesis”, and “IL-2/STAT5 signaling”, which is consistent with the current research on DIO2. Our results emphasize that DIO2 expression is closely related to tumor cells, immune cell infiltration, and TME components, affecting cancer prognosis. These results provide new insights for developing more effective anti-TH treatment.

There are several limitations in this study. First, although the study involved a bioinformatic analysis of DIO2, including its expression, prognostic value, associations with immune cell infiltration, and mutation status in various human cancer types, there were not in vitro or in vivo experiments to validate the results. Therefore, future studies should focus on the mechanisms of DIO2 in various human cancer types. Second, we analyzed prognostic data from TCGA and Kaplan–Meier (KM) plotter and there might be heterogeneity among these datasets. In addition, our bioinformatic analyses are mostly focused on DIO2 gene and mRNA assessment in cancer; however, it is widely demonstrated that the D2 enzymatic activity is subjected to a very complex post-transcriptional regulation and that DIO2 mRNA levels do not perfectly mirror the activity profile, thus additional studies will be essential to validate the D2 protein as a cancer biomarker.

In conclusion, we generated a comprehensive knowledge base of DIO2 as a useful pan-cancer biomarker and uncovered its landscapes, such as DIO2 expression characteristics, prognostic value, mutation profiles, associations with tumor-infiltrating immune cells, and associated molecular pathways across different cancer types. Overall, we have provided new clues for improving cancer diagnosis and developing cancer immunotherapies that target DIO2.

## Supplementary Information

Below is the link to the electronic supplementary material.

Supplementary file1 (DOCX 23 kb)

Supplementary file2 (PDF 17428 kb)

## References

[1] Cheng SY et al (2010) Molecular aspects of thyroid hormone actions. Endocr Rev 31:139–170. 10.1210/er.2009-0007 20051527 10.1210/er.2009-0007 PMC2852208

[2] Brent GA (2012) Mechanisms of thyroid hormone action. J Clin Invest 122:3035–3043. 10.1172/JCI60047 22945636 10.1172/JCI60047 PMC3433956

[3] Mullur R et al (2014) Thyroid hormone regulation of metabolism. Physiol Rev 94:355–382. 10.1152/physrev.00030.2013 24692351 10.1152/physrev.00030.2013 PMC4044302

[4] Dentice M, Salvatore D (2011) Deiodinases: the balance of thyroid hormone: local impact of thyroid hormone inactivation. J Endocrinol 209:273–282. 10.1530/JOE-11-0002 21398344 10.1530/JOE-11-0002

[5] Williams GR, Bassett JH (2011) Deiodinases: the balance of thyroid hormone: local control of thyroid hormone action: role of type 2 deiodinase. J Endocrinol 209:261–272. 10.1530/JOE-10-0448 21292729 10.1530/JOE-10-0448

[6] Kim WG, Cheng SY (2013) Thyroid hormone receptors and cancer. Biochim Biophys Acta 1830:3928–3936. 10.1016/j.bbagen.2012.04.002 22507269 10.1016/j.bbagen.2012.04.002 PMC3406244

[7] Badziong J et al (2017) Differential regulation of monocarboxylate transporter 8 expression in thyroid cancer and hyperthyroidism. Eur J Endocrinol 177:243–250. 10.1530/EJE-17-0279 28576880 10.1530/EJE-17-0279

[8] Nappi A et al (2020) The NANOG Transcription Factor Induces Type 2 Deiodinase Expression and Regulates the Intracellular Activation of Thyroid Hormone in Keratinocyte Carcinomas. Cancers (Basel). 10.3390/cancers12030715 32197405 10.3390/cancers12030715 PMC7140064

[9] Davis PJ et al (2021) Nongenomic Actions of Thyroid Hormone: The Integrin Component. Physiol Rev 101:319–352. 10.1152/physrev.00038.2019 32584192 10.1152/physrev.00038.2019

[10] Cicatiello AG et al (2017) Thyroid hormone promotes differentiation of colon cancer stem cells. Mol Cell Endocrinol 459:84–89. 10.1016/j.mce.2017.03.017 28342853 10.1016/j.mce.2017.03.017

[11] Miro C et al (2017) The Concerted Action of Type 2 and Type 3 Deiodinases Regulates the Cell Cycle and Survival of Basal Cell Carcinoma Cells. Thyroid 27:567–576. 10.1089/thy.2016.0532 28088877 10.1089/thy.2016.0532

[12] Luongo C et al (2014) The sonic hedgehog-induced type 3 deiodinase facilitates tumorigenesis of basal cell carcinoma by reducing Gli2 inactivation. Endocrinology 155:2077–2088. 10.1210/en.2013-2108 24693967 10.1210/en.2013-2108 PMC5393316

[13] Miro C et al (2021) Thyroid Hormone Enhances Angiogenesis and the Warburg Effect in Squamous Cell Carcinomas. Cancers (Basel). 10.3390/cancers13112743 34205977 10.3390/cancers13112743 PMC8199095

[14] Khan SR et al (2016) Thyroid Function and Cancer Risk: The Rotterdam Study. J Clin Endocrinol Metab 101:5030–5036. 10.1210/jc.2016-2104 27648963 10.1210/jc.2016-2104

[15] Dentice M et al (2007) Sonic hedgehog-induced type 3 deiodinase blocks thyroid hormone action enhancing proliferation of normal and malignant keratinocytes. Proc Natl Acad Sci U S A 104:14466–14471. 10.1073/pnas.0706754104 17720805 10.1073/pnas.0706754104 PMC1964817

[16] Di Girolamo D et al (2016) Reciprocal interplay between thyroid hormone and microRNA-21 regulates hedgehog pathway-driven skin tumorigenesis. J Clin Invest 126:2308–2320. 10.1172/JCI84465 27159391 10.1172/JCI84465 PMC4887175

[17] Di Cicco E et al (2021) Germ Line Mutations in the Thyroid Hormone Receptor Alpha Gene Predispose to Cutaneous Tags and Melanocytic Nevi. Thyroid 31:1114–1126. 10.1089/thy.2020.0391 33509032 10.1089/thy.2020.0391 PMC8290313

[18] Epstein EH (2008) Basal cell carcinomas: attack of the hedgehog. Nat Rev Cancer 8:743–754. 10.1038/nrc2503 18813320 10.1038/nrc2503 PMC4457317

[19] Alam M, Ratner D (2001) Cutaneous squamous-cell carcinoma. N Engl J Med 344:975–983. 10.1056/NEJM200103293441306 11274625 10.1056/NEJM200103293441306

[20] Miro C et al (2019) Thyroid hormone induces progression and invasiveness of squamous cell carcinomas by promoting a ZEB-1/E-cadherin switch. Nat Commun 10:5410. 10.1038/s41467-019-13140-2 31776338 10.1038/s41467-019-13140-2 PMC6881453

[21] Hepburn AC et al (2019) The induction of core pluripotency master regulators in cancers defines poor clinical outcomes and treatment resistance. Oncogene 38:4412–4424. 10.1038/s41388-019-0712-y 30742096 10.1038/s41388-019-0712-y PMC6546609

[22] Miro C et al (2022) Thyroid hormone and androgen signals mutually interplay and enhance inflammation and tumorigenic activation of tumor microenvironment in prostate cancer. Cancer Lett 532:215581. 10.1016/j.canlet.2022.215581 35134514 10.1016/j.canlet.2022.215581

[23] Torabinejad S et al (2023) The androgen-thyroid hormone crosstalk in prostate cancer and the clinical implications. Eur Thyroid J. 10.1530/ETJ-22-0228 36930264 10.1530/ETJ-22-0228 PMC10160561

[24] Mariani A et al (2019) Genes associated with bowel metastases in ovarian cancer. Gynecol Oncol 154:495–504. 10.1016/j.ygyno.2019.06.010 31204077 10.1016/j.ygyno.2019.06.010 PMC8767766

[25] Kojima Y et al (2019) Stromal iodothyronine deiodinase 2 (DIO2) promotes the growth of intestinal tumors in Apc(Delta716) mutant mice. Cancer Sci 110:2520–2528. 10.1111/cas.14100 31215118 10.1111/cas.14100 PMC6676103

[26] Nappi A et al (2023) Loss of p53 activates thyroid hormone via type 2 deiodinase and enhances DNA damage. Nat Commun 14:1244. 10.1038/s41467-023-36755-y 36871014 10.1038/s41467-023-36755-y PMC9985592

[27] Li B et al (2016) Comprehensive analyses of tumor immunity: implications for cancer immunotherapy. Genome Biol 17:174. 10.1186/s13059-016-1028-7 27549193 10.1186/s13059-016-1028-7 PMC4993001

[28] Li T et al (2017) TIMER: A Web Server for Comprehensive Analysis of Tumor-Infiltrating Immune Cells. Cancer Res 77:e108–e110. 10.1158/0008-5472.CAN-17-0307 29092952 10.1158/0008-5472.CAN-17-0307 PMC6042652

[29] Li T et al (2020) TIMER2.0 for analysis of tumor-infiltrating immune cells. Nucleic Acids Res 48:W509–W514. 10.1093/nar/gkaa407 32442275 10.1093/nar/gkaa407 PMC7319575

[30] Tang Z et al (2019) GEPIA2: an enhanced web server for large-scale expression profiling and interactive analysis. Nucleic Acids Res 47:W556–W560. 10.1093/nar/gkz430 31114875 10.1093/nar/gkz430 PMC6602440

[31] Cerami E et al (2012) The cBio cancer genomics portal: an open platform for exploring multidimensional cancer genomics data. Cancer Discov 2:401–404. 10.1158/2159-8290.CD-12-0095 22588877 10.1158/2159-8290.CD-12-0095 PMC3956037

[32] Gao J et al (2013) Integrative analysis of complex cancer genomics and clinical profiles using the cBioPortal. Sci Signal. 10.1126/scisignal.2004088 23550210 10.1126/scisignal.2004088 PMC4160307

[33] Chandrashekar DS et al (2017) UALCAN: A Portal for Facilitating Tumor Subgroup Gene Expression and Survival Analyses. Neoplasia 19:649–658. 10.1016/j.neo.2017.05.002 28732212 10.1016/j.neo.2017.05.002 PMC5516091

[34] Ru B et al (2019) TISIDB: an integrated repository portal for tumor-immune system interactions. Bioinformatics 35:4200–4202. 10.1093/bioinformatics/btz210 30903160 10.1093/bioinformatics/btz210

[35] Chen EY et al (2013) Enrichr: interactive and collaborative HTML5 gene list enrichment analysis tool. BMC Bioinformatics 14:128. 10.1186/1471-2105-14-128 23586463 10.1186/1471-2105-14-128 PMC3637064

[36] Kuleshov MV et al (2016) Enrichr: a comprehensive gene set enrichment analysis web server 2016 update. Nucleic Acids Res 44:W90-97. 10.1093/nar/gkw377 27141961 10.1093/nar/gkw377 PMC4987924

[37] Xie Z et al (2021) Gene Set Knowledge Discovery with Enrichr. Curr Protoc 1:e90. 10.1002/cpz1.90 33780170 10.1002/cpz1.90 PMC8152575

[38] Zhou Y et al (2019) Metascape provides a biologist-oriented resource for the analysis of systems-level datasets. Nat Commun 10:1523. 10.1038/s41467-019-09234-6 30944313 10.1038/s41467-019-09234-6 PMC6447622

[39] Mina M et al (2017) Conditional Selection of Genomic Alterations Dictates Cancer Evolution and Oncogenic Dependencies. Cancer Cell 32(155–168):e156. 10.1016/j.ccell.2017.06.010 10.1016/j.ccell.2017.06.010 28756993

[40] Martinez-Jimenez F et al (2023) Pan-cancer whole-genome comparison of primary and metastatic solid tumours. Nature 618:333–341. 10.1038/s41586-023-06054-z 37165194 10.1038/s41586-023-06054-z PMC10247378

[41] Zeold A et al (2006) Characterization of the nuclear factor-kappa B responsiveness of the human dio2 gene. Endocrinology 147:4419–4429. 10.1210/en.2005-1608 16728495 10.1210/en.2005-1608

[42] Janowska M et al (2022) An Assessment of GPX1 (rs1050450), DIO2 (rs225014) and SEPP1 (rs7579) Gene Polymorphisms in Women with Endometrial Cancer. Genes (Basel). 10.3390/genes13020188 35205233 10.3390/genes13020188 PMC8871918

[43] Gawandi S et al (2024) Determination of frequency of type 2 deiodinase Thr92Ala polymorphism (rs225014) in (131)I-treated differentiated thyroid cancer patients undertaking L-thyroxine (L-T4) suppression therapy. Indian J Nucl Med 39:24–28. 10.4103/ijnm.ijnm_120_23 38817730 10.4103/ijnm.ijnm_120_23 PMC11135370

[44] Kulis M, Esteller M (2010) DNA methylation and cancer. Adv Genet 70:27–56. 10.1016/B978-0-12-380866-0.60002-2 20920744 10.1016/B978-0-12-380866-0.60002-2

[45] Delpu Y et al (2013) DNA methylation and cancer diagnosis. Int J Mol Sci 14:15029–15058. 10.3390/ijms140715029 23873296 10.3390/ijms140715029 PMC3742286

[46] Morgan AE et al (2018) The role of DNA methylation in ageing and cancer. Proc Nutr Soc 77:412–422. 10.1017/S0029665118000150 29708096 10.1017/S0029665118000150

