# Supplementary material for: The thyroid hormone activating enzyme, DIO2, is a potential pan-cancer biomarker and immunotherapy target
Source: J Endocrinol Invest. 2025 Jan 17;48(5):1149–72. doi: 10.1007/s40618-024-02526-9 (PMC12049402; doi:10.1007/s40618-024-02526-9)
Supplement: Supplementary file 1 — Supplementary file1 (DOCX 23 kb) [file 40618_2024_2526_MOESM1_ESM.docx]

**Supplemental Figure 1. Correlation analysis between DIO2 expression and immune infiltration of Cancer-Associated Fibroblasts (CAFs).** Different algorithms (EPIC, MCP-COUNTER, TIDE and XCELL included into the “Immune-Gene” module of the TIMER2.0 (Tumor IMmune Estimation Resource, version 2.0, <http://timer.cistrome.org/>) were used to explore the potential correlation between the expression level of the DIO2 gene and the infiltration level of Cancer-Associated Fibroblasts (CAFs) across BLCA, BRCA, COAD, LUAD, LUSC and OV.

**Supplemental Figure 2. Correlation analysis between DIO2 expression and immune infiltration of Cancer-Associated Fibroblasts (CAFs).** Different algorithms (EPIC, MCP-COUNTER, TIDE and XCELL included into the “Immune-Gene” module of the TIMER2.0 (Tumor IMmune Estimation Resource, version 2.0, <http://timer.cistrome.org/>) were used to explore the potential correlation between the expression level of the DIO2 gene and the infiltration level of Cancer-Associated Fibroblasts (CAFs) across PAAD, READ, SARC, STAD, UCEC and UCS.

**Supplemental Figure 3. Correlation analysis between DIO2 expression and immune infiltration of Dendritic Cells (DCs).** Different algorithms (TIMER, MCP-COUNTER and XCELL included into the “Immune-Gene” module of the TIMER2.0 (Tumor IMmune Estimation Resource, version 2.0, <http://timer.cistrome.org/>) were used to explore the potential correlation between the expression level of the DIO2 gene and the infiltration level of Dendritic Cells (DCs) across BLCA, BRCA, COAD, LUAD, LUSC and OV.

**Supplemental Figure 4. Correlation analysis between DIO2 expression and immune infiltration of Dendritic Cells (DCs).** Different algorithms (TIMER, MCP-COUNTER and XCELL included into the “Immune-Gene” module of the TIMER2.0 (Tumor IMmune Estimation Resource, version 2.0, <http://timer.cistrome.org/>) were used to explore the potential correlation between the expression level of the DIO2 gene and the infiltration level of Dendritic Cells (DCs) across PAAD, READ, SARC, STAD, UCEC and UCS.

**Supplemental Figure 5. Correlation analysis between DIO2 expression and immune infiltration of Endothelial Cells (ECs).** Different algorithms (EPIC, MCP-COUNTER and XCELL included into the “Immune-Gene” module of the TIMER2.0 (Tumor IMmune Estimation Resource, version 2.0, <http://timer.cistrome.org/>) were used to explore the potential correlation between the expression level of the DIO2 gene and the infiltration level of Endothelial Cells (ECs) across BLCA, BRCA, COAD, LUAD, LUSC and OV.

**Supplemental Figure 6. Correlation analysis between DIO2 expression and immune infiltration of Endothelial Cells (ECs).** Different algorithms (EPIC, MCP-COUNTER and XCELL included into the “Immune-Gene” module of the TIMER2.0 (Tumor IMmune Estimation Resource, version 2.0, <http://timer.cistrome.org/>) were used to explore the potential correlation between the expression level of the DIO2 gene and the infiltration level of Endothelial Cells (ECs) across PAAD, READ, SARC, STAD, UCEC and UCS.

**Supplemental Figure 7. Correlation analysis between DIO2 expression and immune infiltration of Tumor-Associated Macrophages (TAMs).** Different algorithms (EPIC, MCP-COUNTER, TIMER and XCELL included into the “Immune-Gene” module of the TIMER2.0 (Tumor IMmune Estimation Resource, version 2.0, <http://timer.cistrome.org/>) were used to explore the potential correlation between the expression level of the DIO2 gene and the infiltration level of Tumor-Associated Macrophages (TAMs) across BLCA, BRCA, COAD, LUAD, LUSC and OV.

**Supplemental Figure 8. Correlation analysis between DIO2 expression and immune infiltration of Tumor-Associated Macrophages (TAMs).** Different algorithms (EPIC, MCP-COUNTER, TIMER and XCELL included into the “Immune-Gene” module of the TIMER2.0 (Tumor IMmune Estimation Resource, version 2.0, <http://timer.cistrome.org/>) were used to explore the potential correlation between the expression level of the DIO2 gene and the infiltration level of Tumor-Associated Macrophages (TAMs) across PAAD, READ, SARC, STAD, UCEC and UCS.

**Supplemental Figure 9. Correlation analysis between DIO2 expression and immune infiltration of CD4^+^ T-cell.** Different algorithms (EPIC, TIMER and XCELL included into the “Immune-Gene” module of the TIMER2.0 (Tumor IMmune Estimation Resource, version 2.0, <http://timer.cistrome.org/>) were used to explore the potential correlation between the expression level of the DIO2 gene and the infiltration level of CD4^+^ T-cell across BLCA, BRCA, COAD, LUAD, LUSC and OV.

**Supplemental Figure 10. Correlation analysis between DIO2 expression and immune infiltration of CD4^+^ T-cell.** Different algorithms (EPIC, TIMER and XCELL included into the “Immune-Gene” module of the TIMER2.0 (Tumor IMmune Estimation Resource, version 2.0, <http://timer.cistrome.org/>) were used to explore the potential correlation between the expression level of the DIO2 gene and the infiltration level of CD4^+^ T-cell across PAAD, READ, SARC, STAD, UCEC and UCS.

**Supplemental Figure 11. Correlation analysis between DIO2 expression and immune infiltration of CD8^+^ T-cell.** Different algorithms (EPIC, MCP-COUNTER, TIMER and XCELL included into the “Immune-Gene” module of the TIMER2.0 (Tumor IMmune Estimation Resource, version 2.0, <http://timer.cistrome.org/>) were used to explore the potential correlation between the expression level of the DIO2 gene and the infiltration level of CD8^+^ T-cell across BLCA, BRCA, COAD, LUAD, LUSC and OV.

**Supplemental Figure 12. Correlation analysis between DIO2 expression and immune infiltration of CD8^+^ T-cell.** Different algorithms (EPIC, MCP-COUNTER, TIMER and XCELL included into the “Immune-Gene” module of the TIMER2.0 (Tumor IMmune Estimation Resource, version 2.0, <http://timer.cistrome.org/>) were used to explore the potential correlation between the expression level of the DIO2 gene and the infiltration level of CD8^+^ T-cell across PAAD, READ, SARC, STAD, UCEC and UCS.

**Supplemental Figure 13. Correlations of DIO2 expression with the expression of immunomodulators in the TISIDB database. (A-C)** Correlations between the expression of DIO2 and (A) Immunostimulator, (B) Chemokines and (C) Chemokine Receptors in the TISIDB Database (<http://cis.hku.hk/TISIDB/>). Red and blue represent positive and negative correlations, respectively.

**Supplemental Figure 14. Correlation analysis between DIO2 and NF-kB expression in pan-cancer.** Correlation analyses were analyzed by Similar Genes Detection tool of GEPIA2 and computed by Spearman’s rank correlation test (Spearman correlation coefficient, R). Significant positive correlation (p-value < 0.05, R > 0).
