# Supplementary material for: The thyroid hormone activating enzyme, DIO2, is a potential pan-cancer biomarker and immunotherapy target
Source: J Endocrinol Invest. 2025 Jan 17;48(5):1149–72. doi: 10.1007/s40618-024-02526-9 (PMC12049402; doi:10.1007/s40618-024-02526-9)
Supplement: Supplementary file 2 — Supplementary file2 (PDF 17428 kb) [file 40618_2024_2526_MOESM2_ESM.pdf]

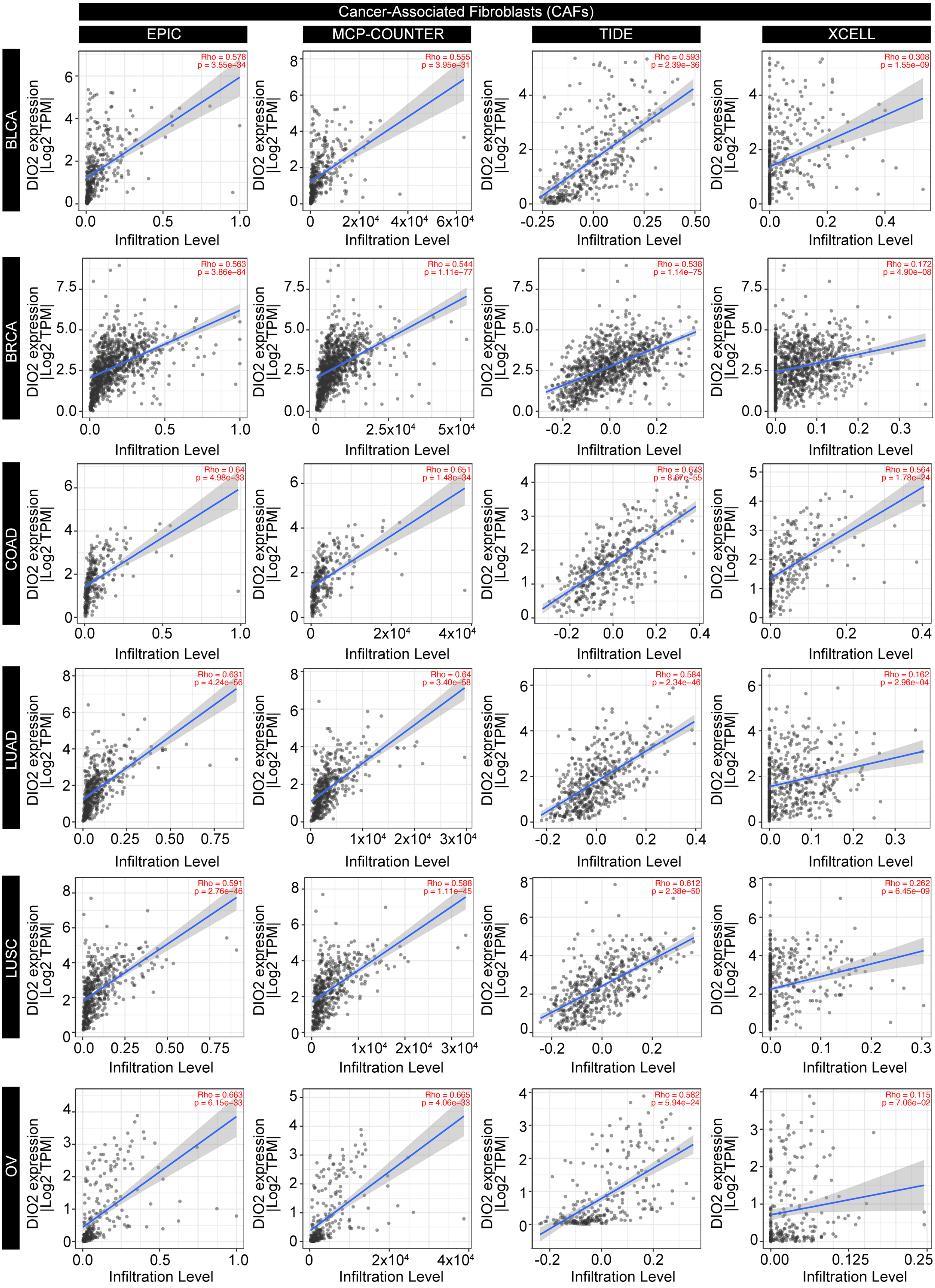

Supplemental Fig. S1

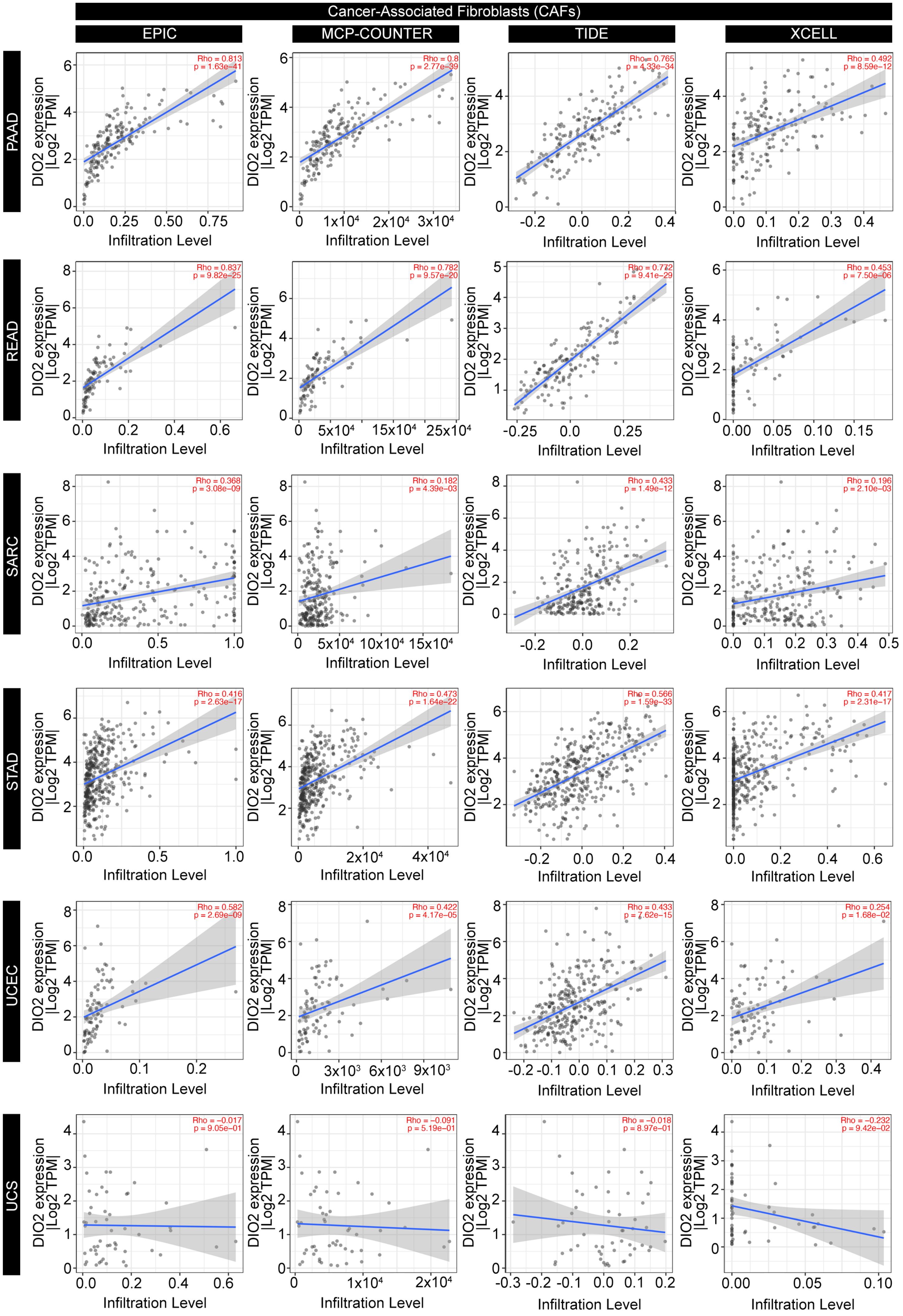

Supplemental Fig. S2

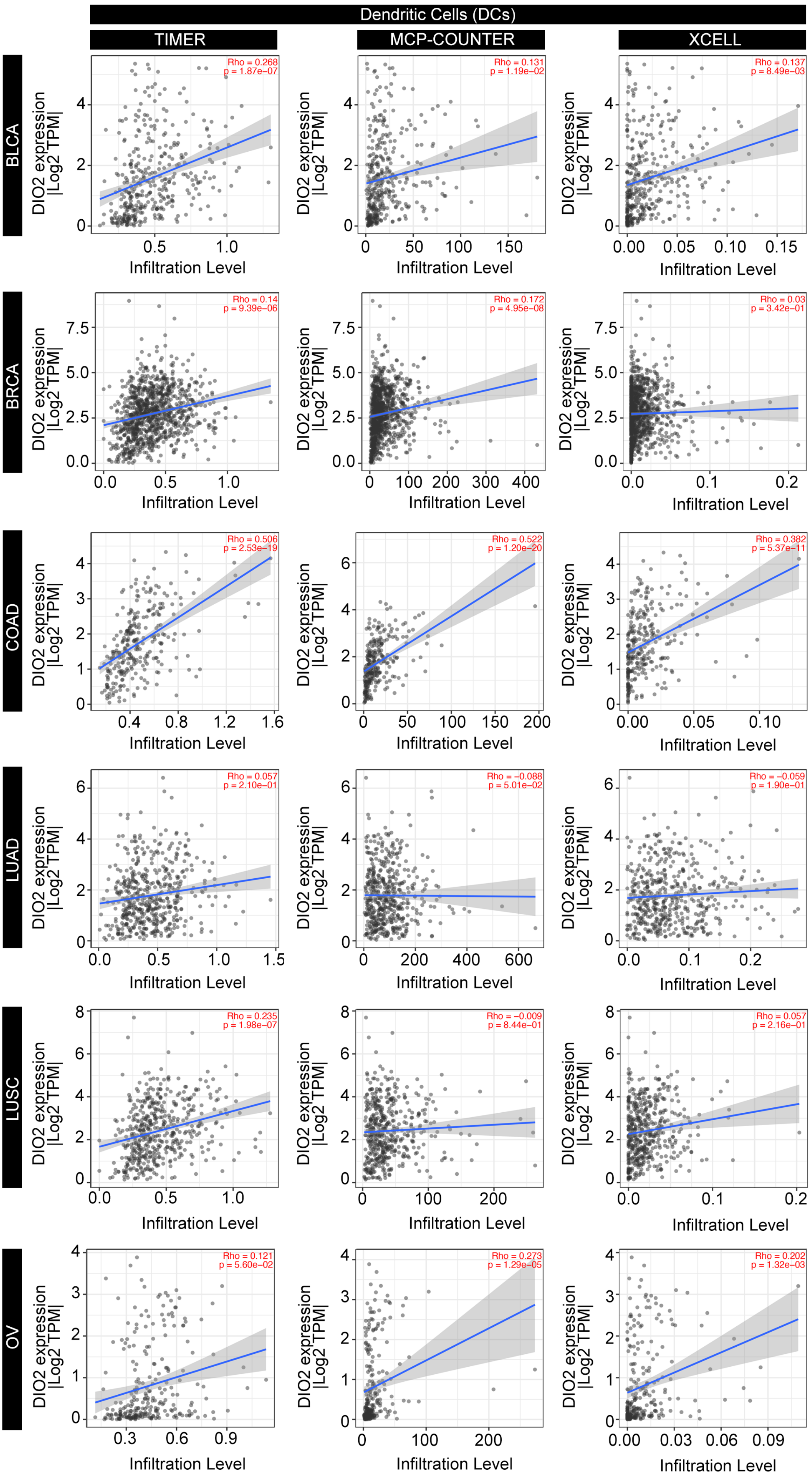

Supplemental Fig. S3

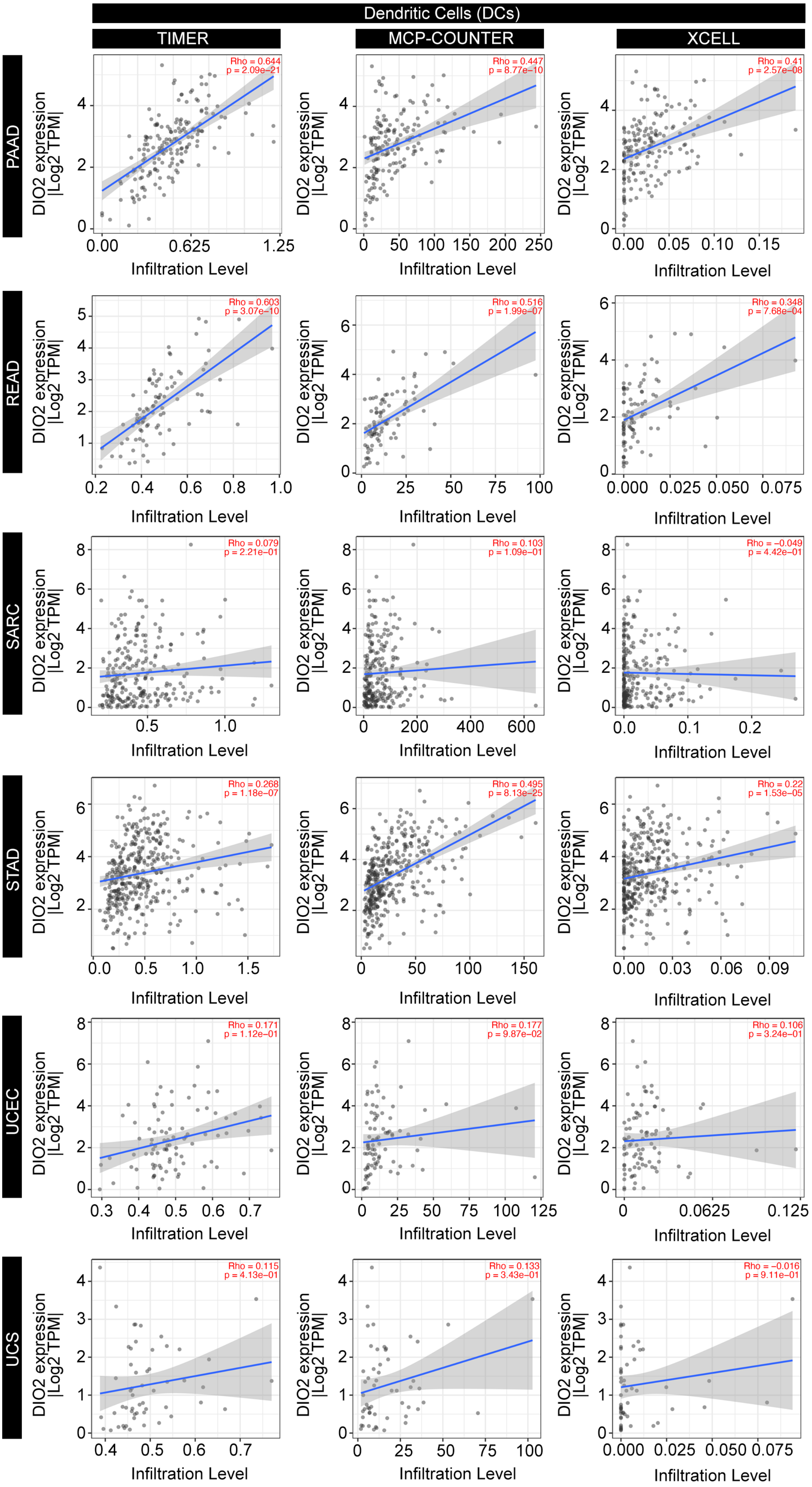

Supplemental Fig. S4

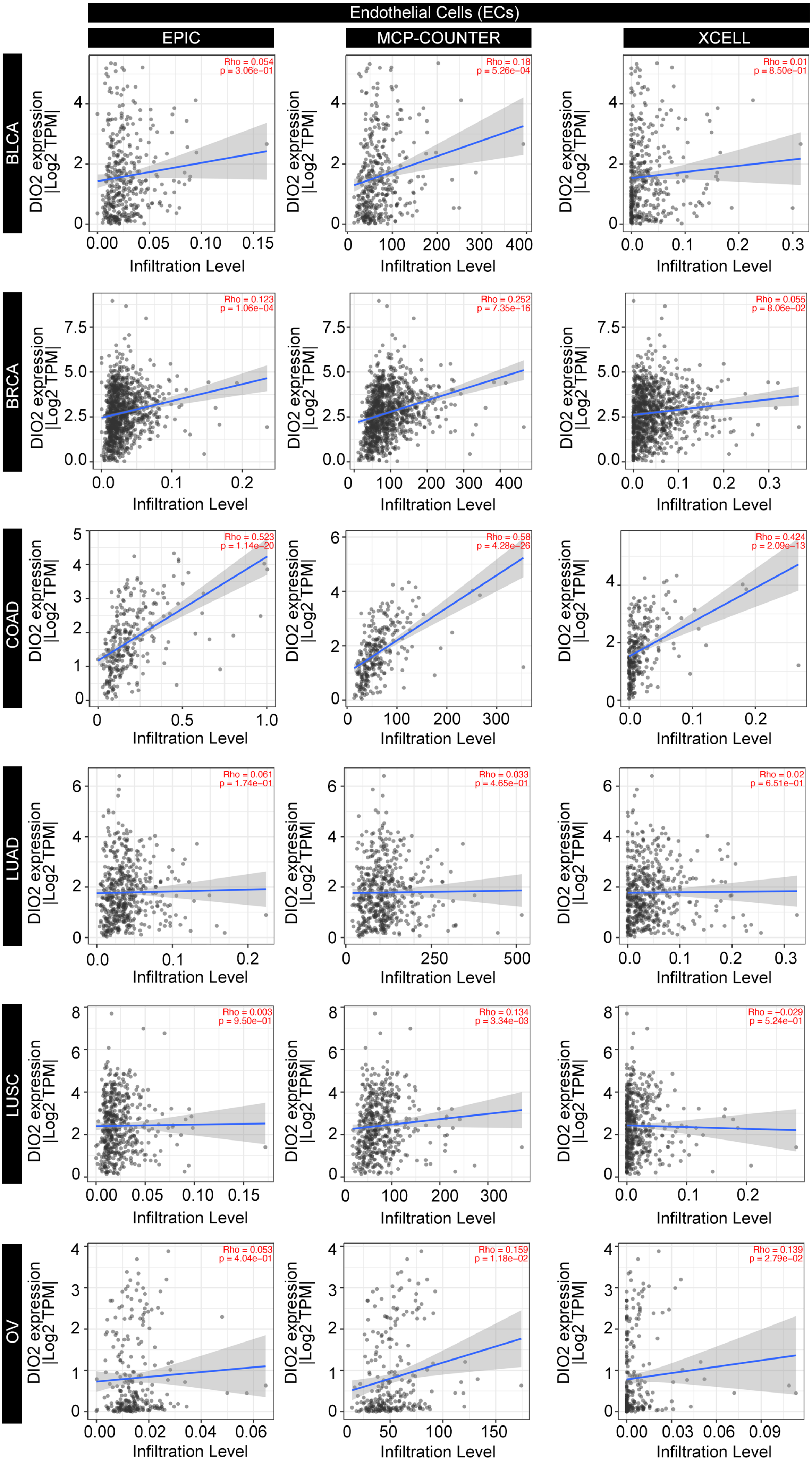

Supplemental Fig. S5

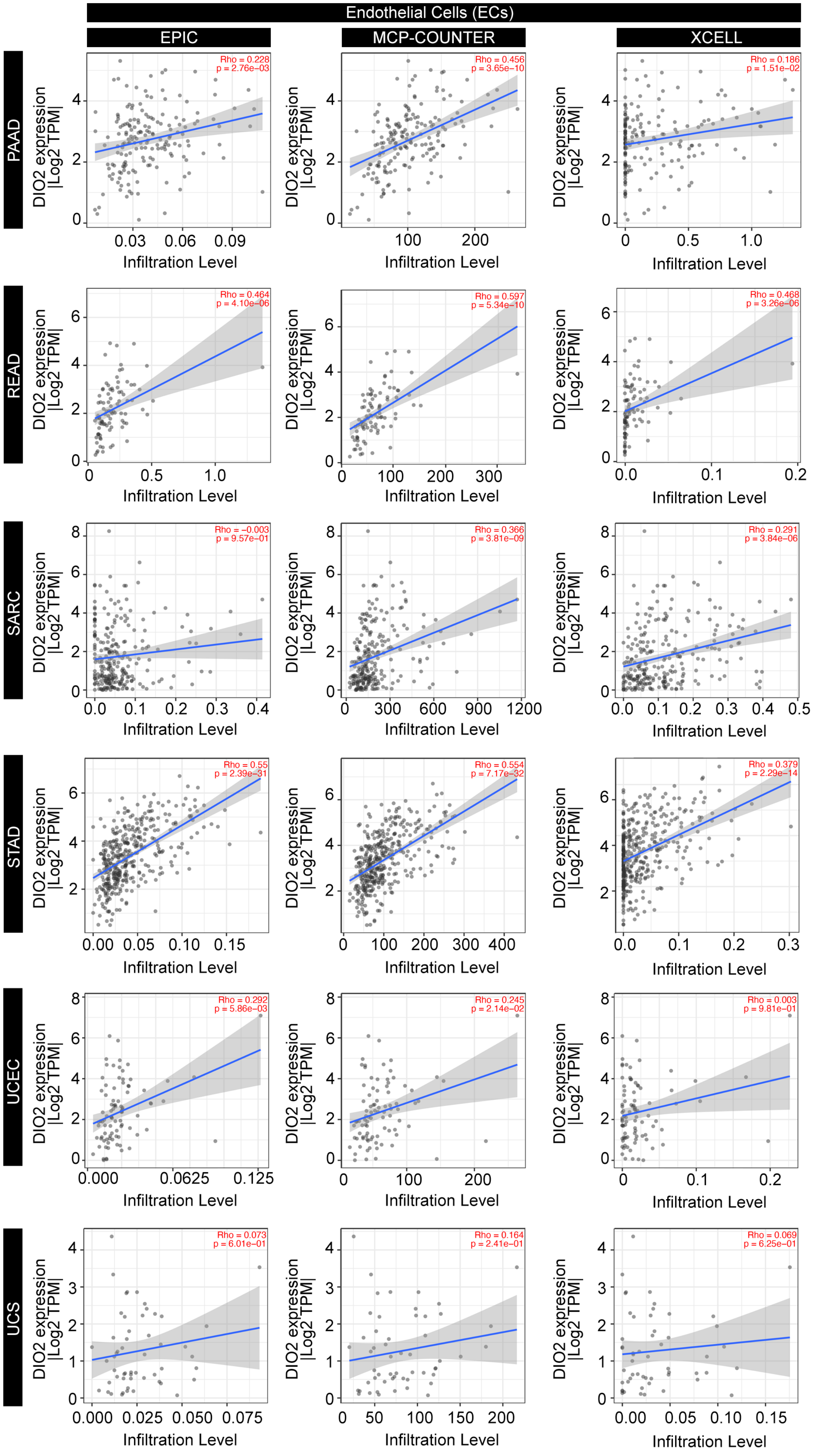

Supplemental Fig. S6

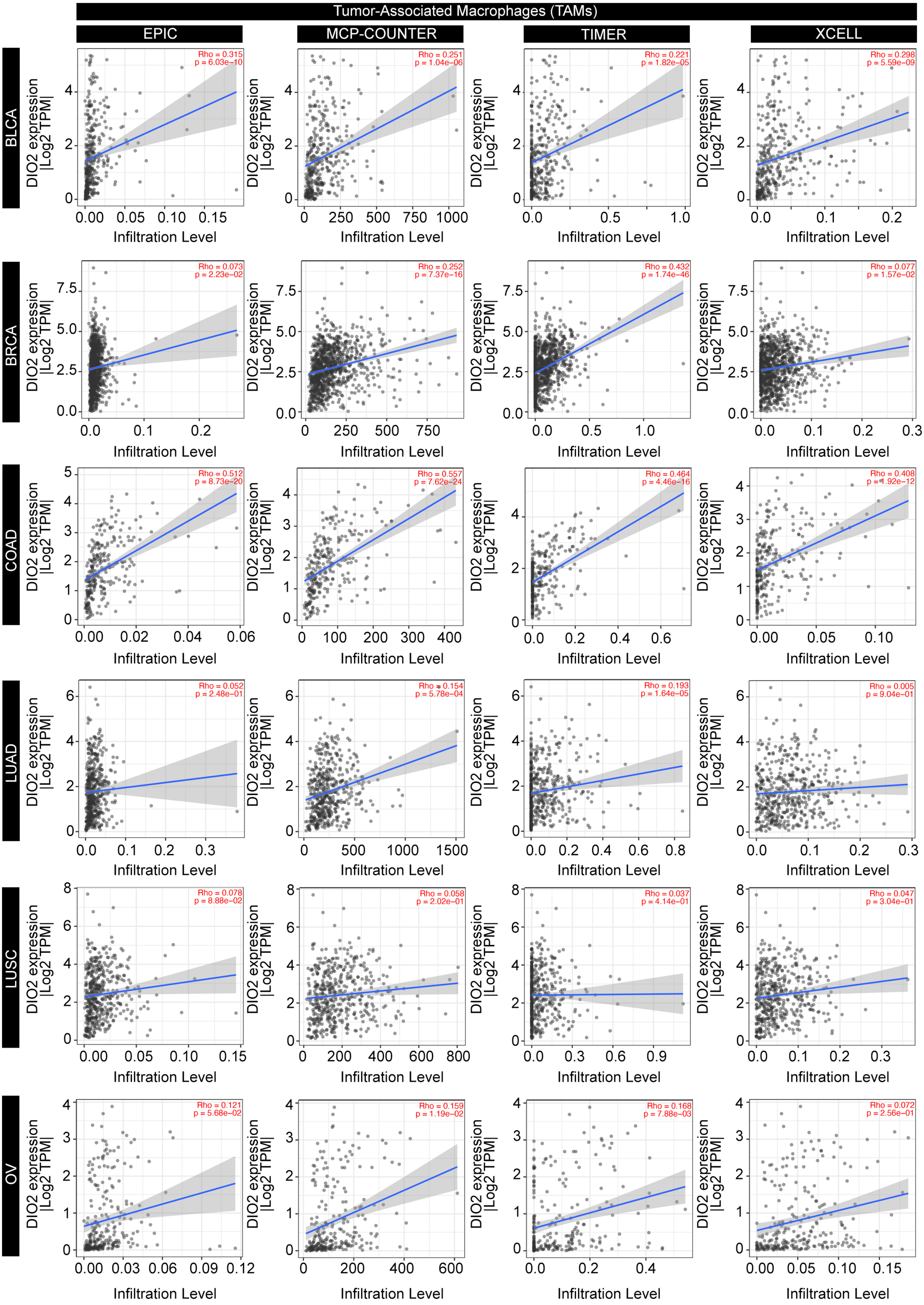

Supplemental Fig. S7

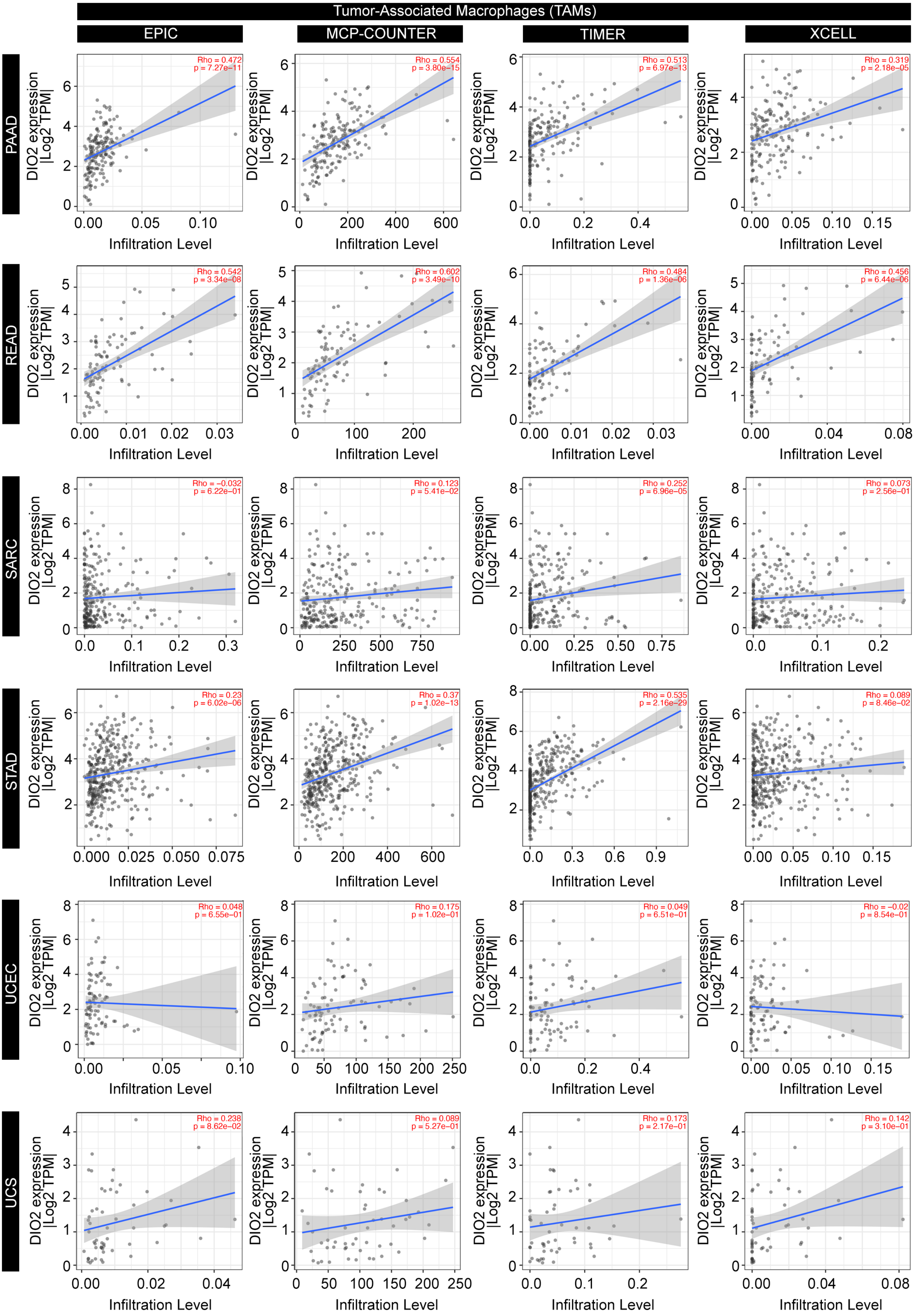

Supplemental Fig. S8

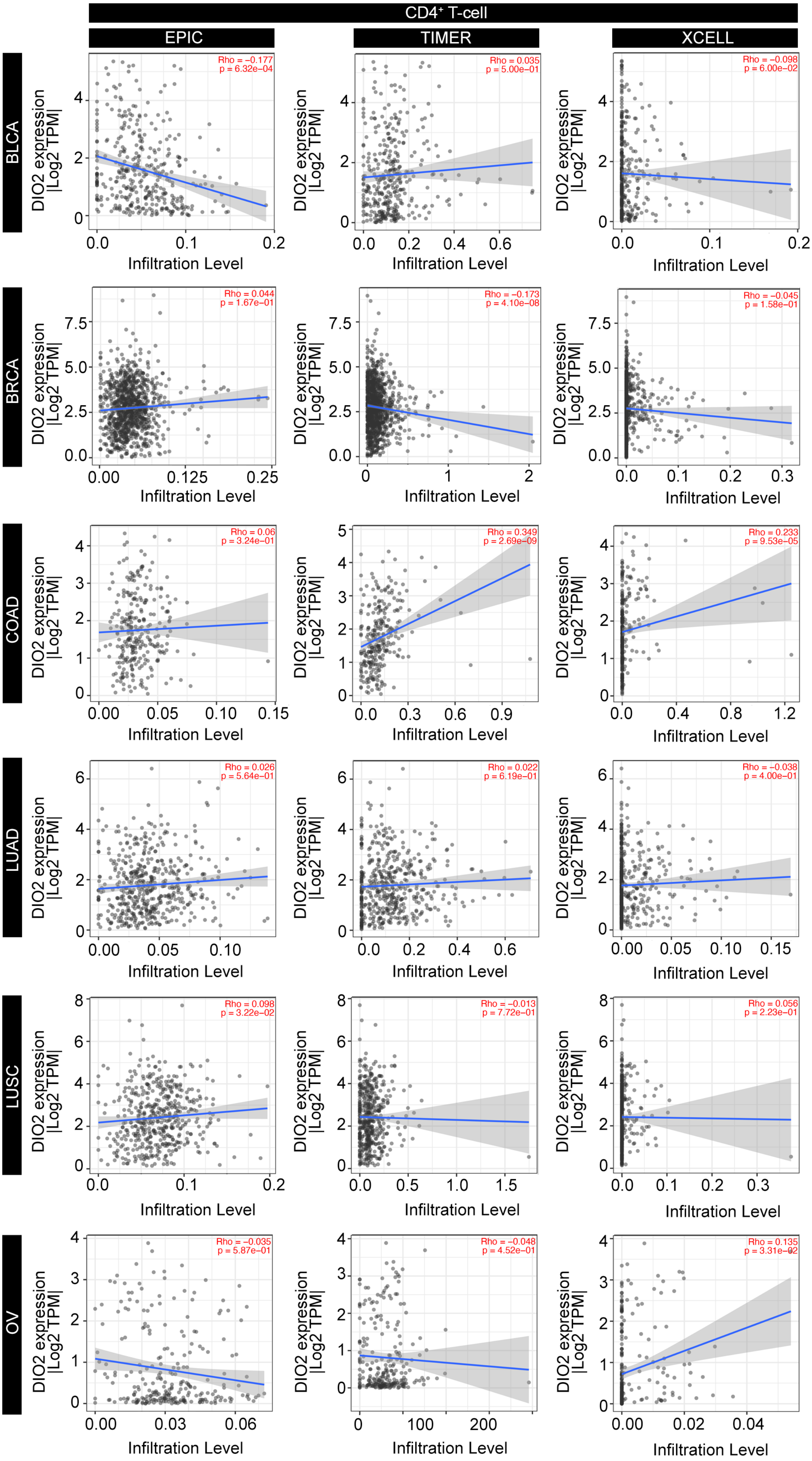

Supplemental Fig. S9

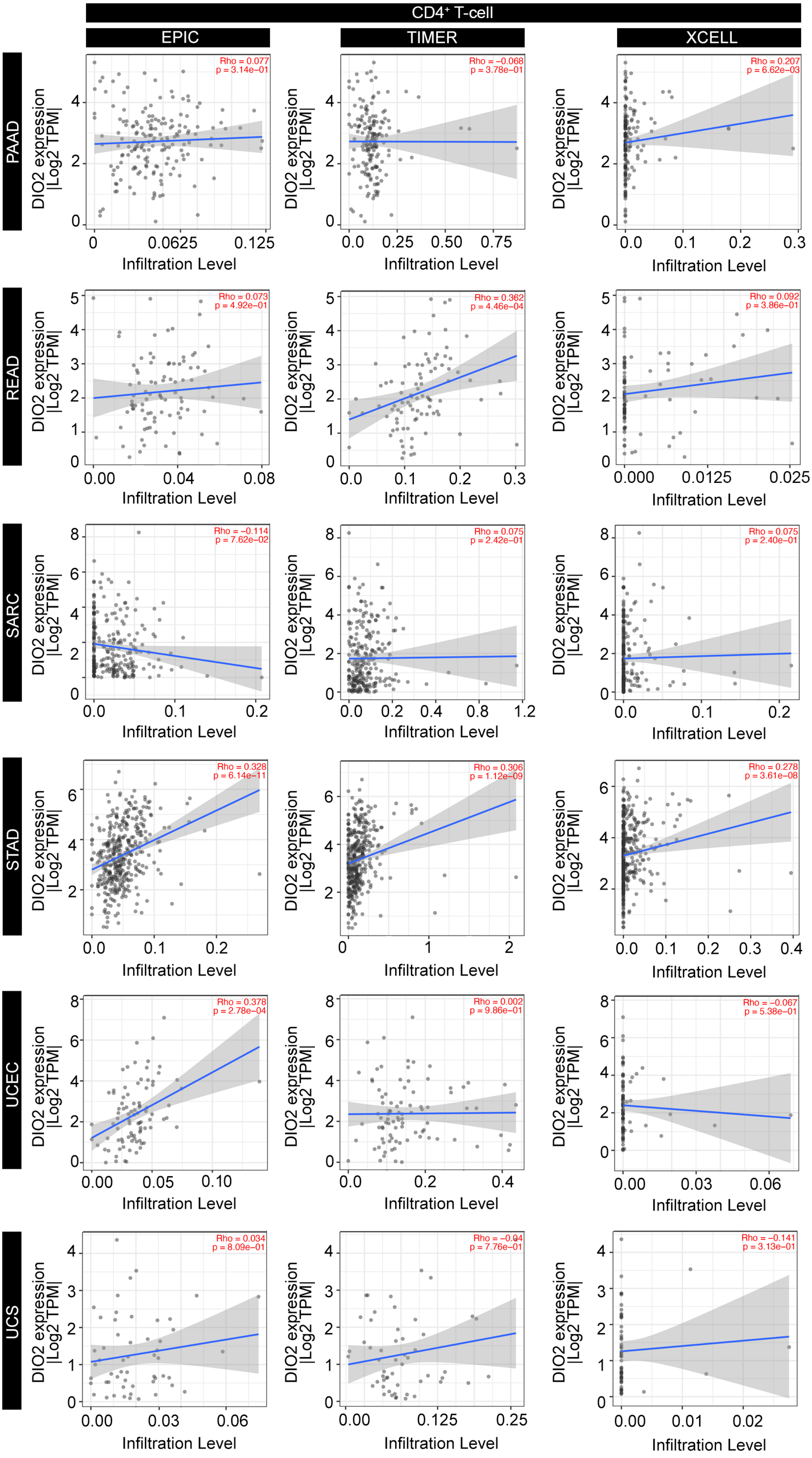

Supplemental Fig. S10

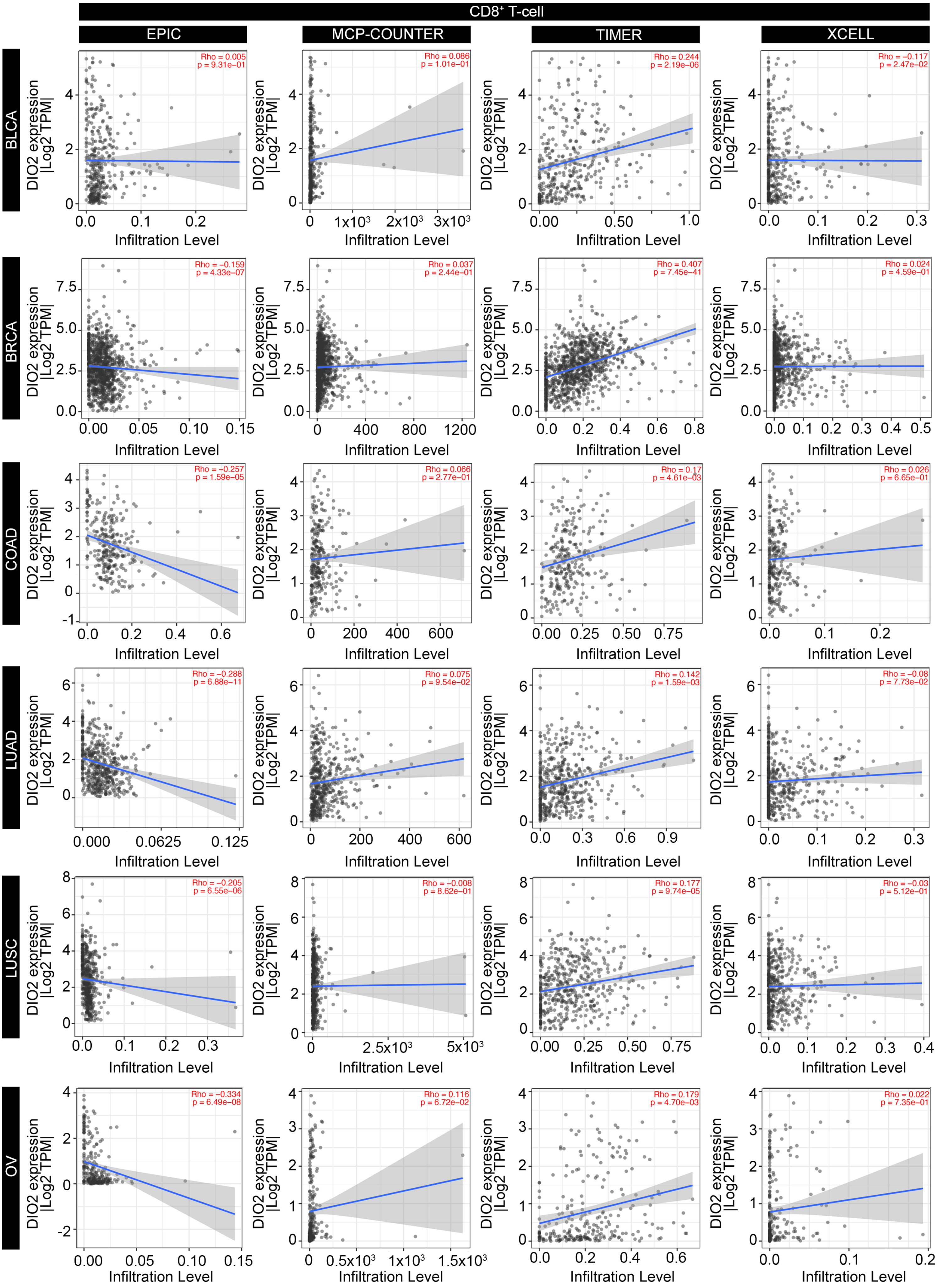

Supplemental Fig. S11

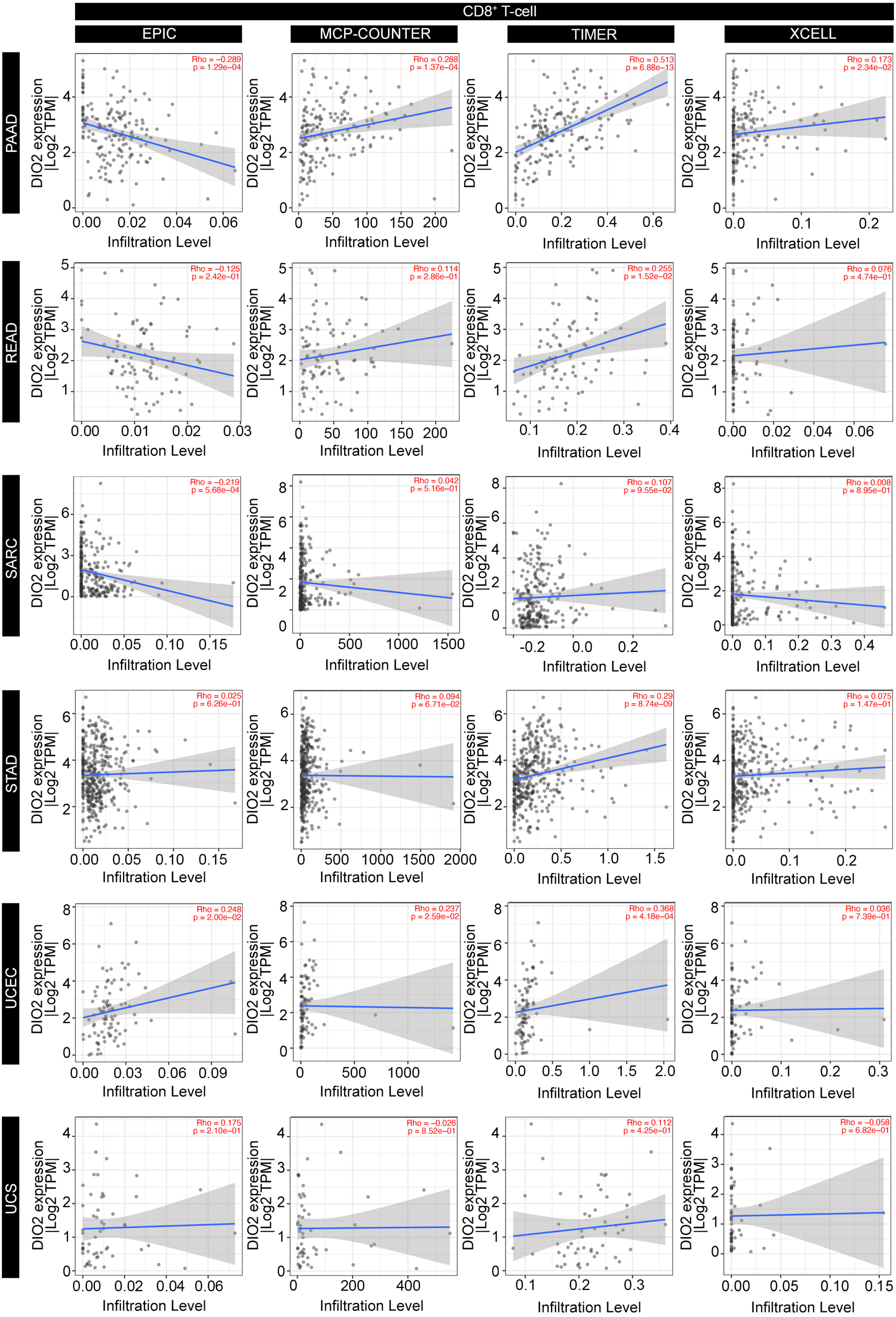

Supplemental Fig. S12

A

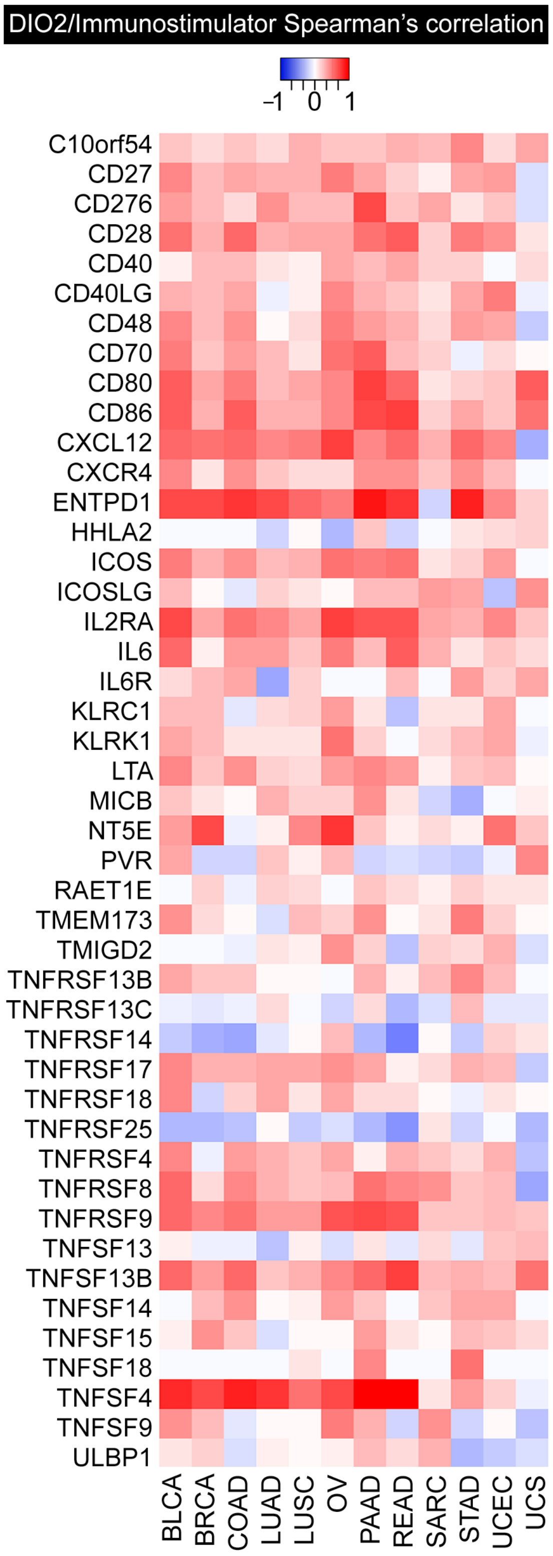

B

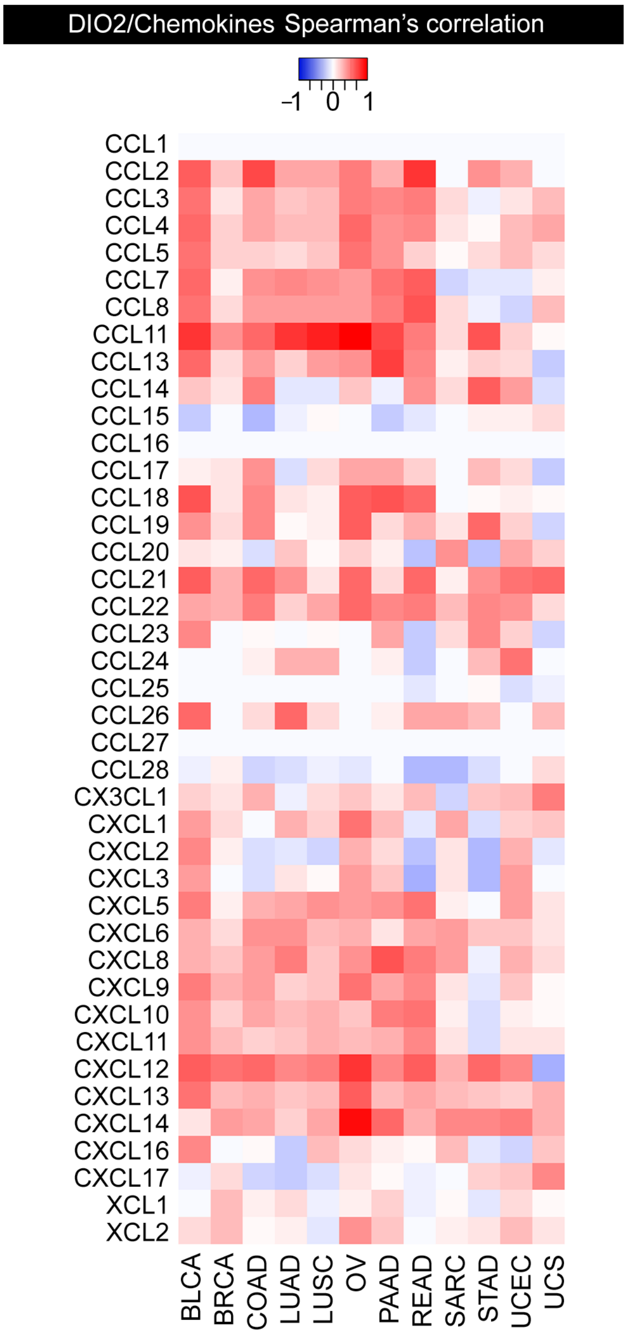

C

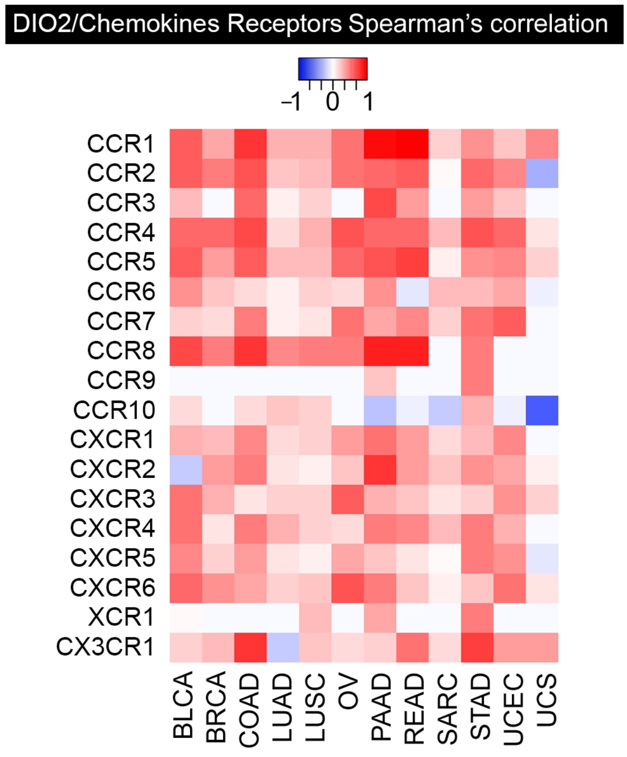

Supplemental Fig. S13

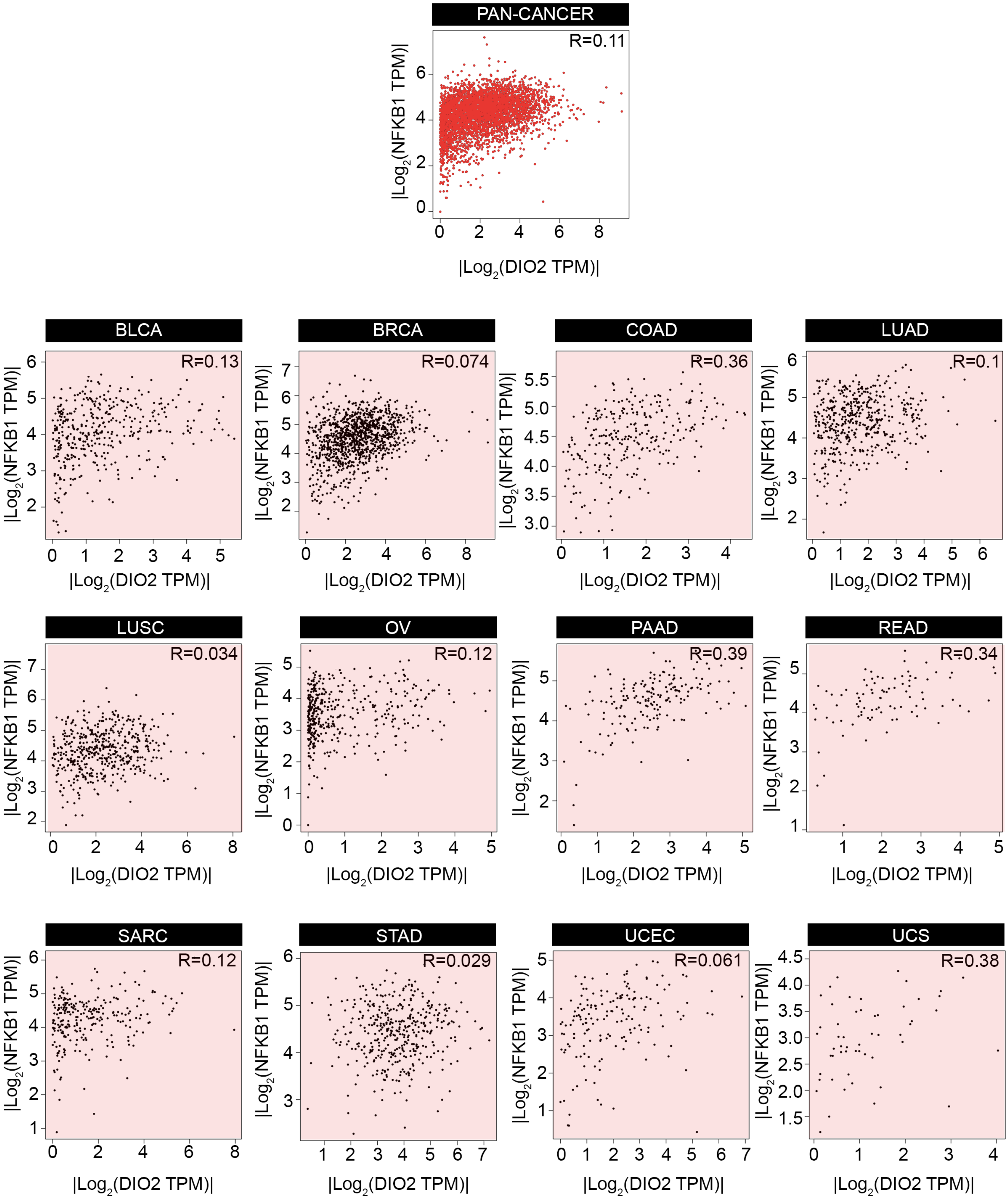

Supplemental Fig. S14
